# The domestication of *Cucurbita argyrosperma* as revealed by the genome of its wild relative

**DOI:** 10.1038/s41438-021-00544-9

**Published:** 2021-05-01

**Authors:** Josué Barrera-Redondo, Guillermo Sánchez-de la Vega, Jonás A. Aguirre-Liguori, Gabriela Castellanos-Morales, Yocelyn T. Gutiérrez-Guerrero, Xitlali Aguirre-Dugua, Erika Aguirre-Planter, Maud I. Tenaillon, Rafael Lira-Saade, Luis E. Eguiarte

**Affiliations:** 1 Departamento de Ecología Evolutiva, Instituto de Ecología, Universidad Nacional Autónoma de México, Circuito Exterior s/n Anexo al Jardín Botánico, 04510 Ciudad de México, México; 2 Department of Ecology and Evolutionary Biology, University of California, Irvine, CA 92697 USA; 3 Departamento de Conservación de la Biodiversidad, El Colegio de la Frontera Sur, Villahermosa, Carretera Villahermosa‐Reforma km 15.5 Ranchería El Guineo 2ª sección, 86280 Villahermosa, Tabasco México; 4 Génétique Quantitative et Evolution – Le Moulon, Université Paris-Saclay, Institut National de Recherche pour l’Agriculture, l’Alimentation et l’Environnement, Centre National de la Recherche Scientifique, AgroParisTech, Gif-sur-Yvette, 91190 France; 5 UBIPRO, Facultad de Estudios Superiores Iztacala, Universidad Nacional Autónoma de México, Av. de los Barrios #1, Col. Los Reyes Iztacala, Tlalnepantla, Edo. de Mex 54090 México

**Keywords:** Population genetics, Genomics, Plant domestication

## Abstract

Despite their economic importance and well-characterized domestication syndrome, the genomic impact of domestication and the identification of variants underlying the domestication traits in *Cucurbita* species (pumpkins and squashes) is currently lacking. *Cucurbita argyrosperma*, also known as cushaw pumpkin or silver-seed gourd, is a Mexican crop consumed primarily for its seeds rather than fruit flesh. This makes it a good model to study *Cucurbita* domestication, as seeds were an essential component of early Mesoamerican diet and likely the first targets of human-guided selection in pumpkins and squashes. We obtained population-level data using tunable Genotype by Sequencing libraries for 192 individuals of the wild and domesticated subspecies of *C. argyrosperma* across Mexico. We also assembled the first high-quality wild *Cucurbita* genome. Comparative genomic analyses revealed several structural variants and presence/absence of genes related to domestication. Our results indicate a monophyletic origin of this domesticated crop in the lowlands of Jalisco. We found evidence of gene flow between the domesticated and wild subspecies, which likely alleviated the effects of the domestication bottleneck. We uncovered candidate domestication genes that are involved in the regulation of growth hormones, plant defense mechanisms, seed development, and germination. The presence of shared selected alleles with the closely related species *Cucurbita moschata* suggests domestication-related introgression between both taxa.

## Introduction

Domestication is an evolutionary process where human societies select, modify and eventually assume control over the reproduction of useful organisms. A mutualistic relationship emerges from this interaction, where humans exploit a particular resource of interest, while the domesticated organism benefits from increased fitness and extended geographical range^1,2^. This is well illustrated in *Cucurbita* L. (pumpkins, squashes, and some gourds), where human-guided domestication and breeding have considerably extended their distribution despite the extinction of their natural dispersers (e.g., mastodons and similar megafauna)^3^. Today, *Cucurbita* stand as successful crops grown and consumed worldwide, with a global annual production of ~24 million tons^4^.

With ca. 21 taxa, the *Cucurbita* genus has experienced independent domestication events in five species^5,6^. Each *Cucurbita* crop experienced a unique selection for specific traits, predominantly defined by the nutritional and cultural needs of early human populations in America^7^. However, many domestication traits are common to domesticated *Cucurbita*, including the loss of bitter compounds (cucurbitacins), the loss of physical defense mechanisms (e.g., urticating trichomes), the loss of seed dormancy, the enlargement of fruits and seeds, and the diversification of fruit morphology^4,8^.

**Fig. 1:**
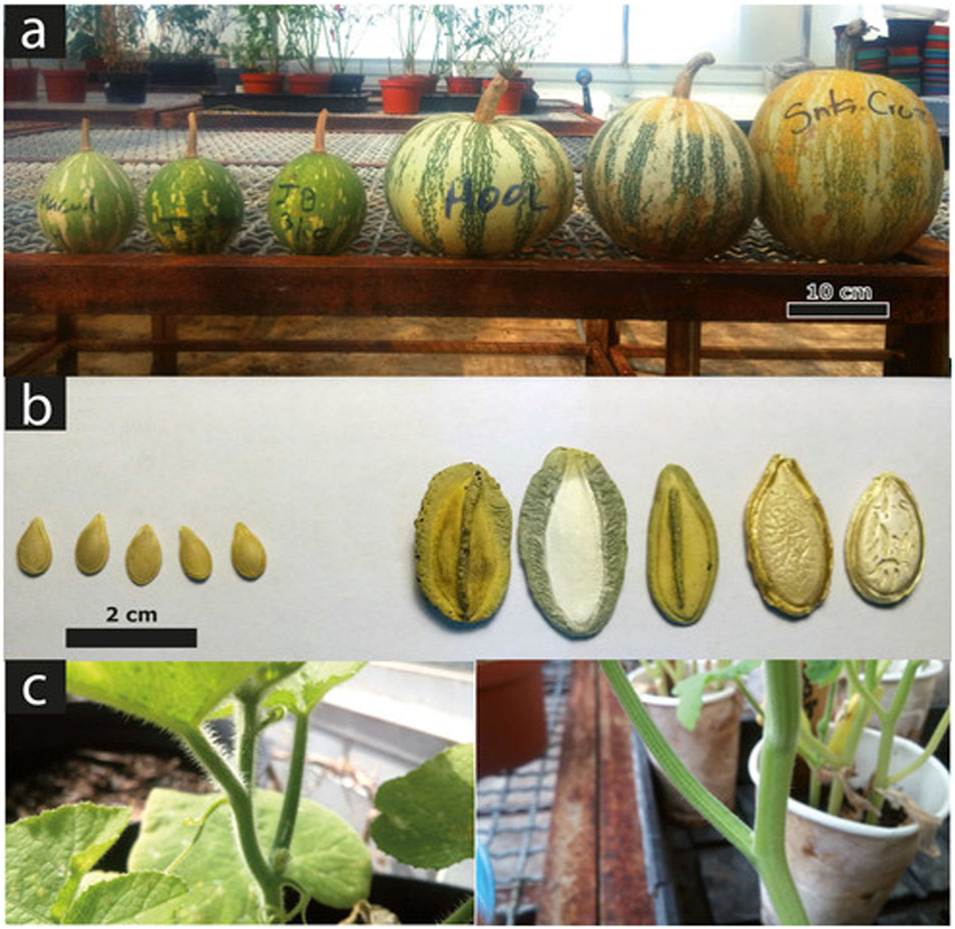
Some morphological differences between *C. argyrosperma* subsp. *sororia* (left) and *C. argyrosperma* subsp. *argyrosperma* (right). Differences in **a** fruit size, **b** seed size and shape, **c** and in the presence of urticating trichomes

The initial steps of *Cucurbita* domestication were most likely directed towards seed rather than flesh consumption^9^. Seeds are rich in both carbohydrates and fatty acids, and cucurbitacins can be removed through boiling and washing; processes that are still employed for the consumption of wild *Cucurbita* seeds in Western Mexico^7^. Because the cultivation of *C. argyrosperma* (pipiana squash, cushaw pumpkin, or silver-seed gourd) is directed towards seed production rather than fruit flesh, it stands as an excellent model to investigate the early steps of *Cucurbita* domestication. *Cucurbita argyrosperma* subsp. *argyrosperma* (*argyrosperma* hereafter) was domesticated in Mesoamerica from its wild relative *Cucurbita argyrosperma* subsp. *sororia* (*sororia* hereafter), according to archaeological and genetic evidence^6,10,11^. *Argyrosperma* exhibits morphological differences from *sororia*, including larger fruits, larger seeds, and lack of urticating trichomes (Fig. 1). The earliest archaeological record of *argyrosperma* is presumed to be from 8700-year-old phytoliths in the Central Balsas Valley (Guerrero), although its taxonomic identity remains uncertain^10^. *C. argyrosperma* is a monoecious outcrossing species and gene flow has been previously described between the domesticated and wild subspecies^12^. Both subspecies are sympatric throughout the Pacific Coast of Mexico and Central America, with a few populations scattered in the coast of the Gulf of Mexico^11,13^. The domesticated taxon is also distributed in the Yucatan Peninsula, where its wild counterpart is absent^11^.

Despite the economic importance and the growing genomic resources in *Cucurbita* species^8,13^, studies aimed at understanding their domestication are still lacking. To start filling this gap, we report here the first genome assembly of the wild relative *sororia*, which complements the existing assembly of *argyrosperma*^14^. The comparison between the genomes allowed us to find genomic structural variants between both subspecies. We characterized a large sample of *argyrosperma* landraces (117 individuals from 19 locations) and *sororia* accessions (50 individuals from 4 locations) using genome-wide data to investigate their demographic history and propose a domestication scenario. We also performed selection scans throughout the genome of *C. argyrosperma* to detect candidate regions associated with the domestication of this species.

## Results

### Genome assembly of *C. argyrosperma* subsp. *sororia*

We sequenced the genome of a wild individual of subspecies *sororia* using Illumina HiSeq4000 (213x coverage) and PacBio Sequel (75.4x coverage). The genome was assembled in 828 contigs with an N50 contig size of 1.3 Mbp and an L50 of 58 contigs (Table S1). A BUSCO analysis^15^ against the *embryophyte odb9* database detected 92.8% of complete BUSCOs, 1.2% fragmented BUSCOs, and 6.0% missing BUSCOs within the genome assembly, similarly to other *Cucurbita* genome assemblies^14,16^. We predicted 30,592 protein-coding genes within the genome assembly using BRAKER2^17^. The BUSCO completeness of the gene predictions (92.5% complete BUSCOs, 3.1% fragmented BUSCOs) is comparable to that of the genome assembly and that of other genome annotations^14,16^, despite using RNA-seq data of a different individual from which the genome was assembled (see “Methods”). Around 35.8% of our *sororia* genome assembly is composed of transposable elements (TEs), slightly higher than the 34.1% of TEs found in a previous *argyrosperma* assembly^14^.

The genome of *argyrosperma* was previously assembled in 920 scaffolds^14^, so we aimed at improving the assemblies for both the *sororia* and the *argyrosperma* genomes using a reference-guided scaffolding step against the genome assembly of *C. moschata*^16^. We anchored 99.97% of the *argyrosperma* genome assembly and 98.8% of the *sororia* genome assembly into 20 pseudomolecules using RaGOO^18^, which corresponds to the haploid chromosome number in *Cucurbita*^19^. Both assemblies show high synteny conservation across the genus (Fig. S1) and confirm a previously reported inversion in chromosome four that is shared with *C. moschata*^16^.

### Structural variants between the wild and domesticated genomes

**Fig. 2:**
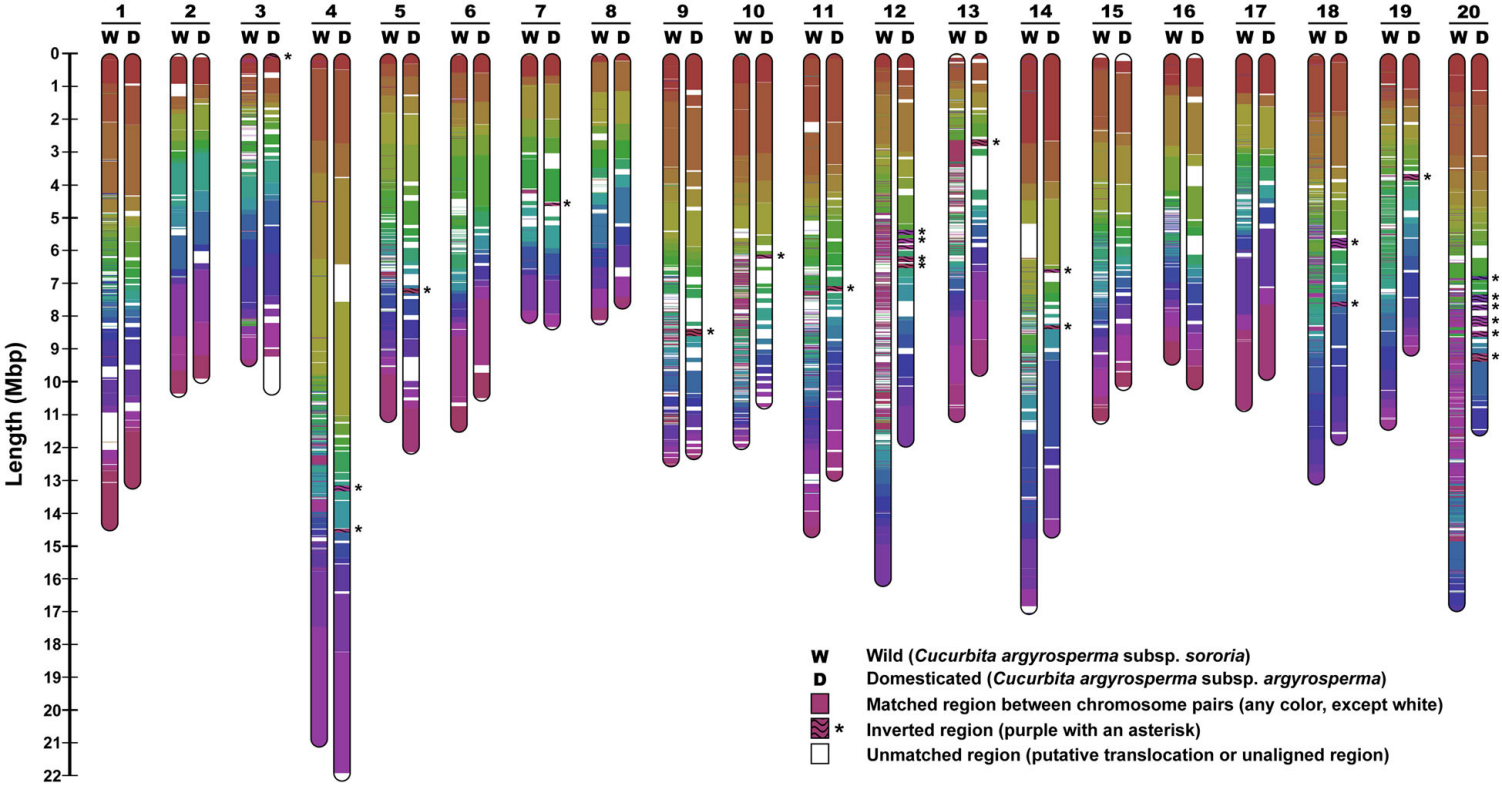
Chromosome map representing the matching regions and putative structural variants between the genome assemblies of *C. argyrosperma* subsp. *sororia* (wild) and *C. argyrosperma* subsp. *argyrosperma* (domesticated). Matching colors represent the aligned homologous regions between both genomes, while white segments represent regions that could not be aligned to the other genome. Inverted regions are highlighted with an asterisk

We compared the genome of *argyrosperma* against the genome of *sororia* (Fig. 2). Some of the centromeres in the genome of *sororia* were larger than in *argyrosperma*, possibly due to a better assembly of the repetitive regions. We found 443 high-confidence structural variants (SVs) such as copy-number variants (CNVs), inversions, and translocations between the wild and the domesticated genomes (Table S2). We also found several regions that could not be aligned between both genomes (Table S2). The size of the *sororia* genome assembly is ~254 Mbp, 9.23% larger than the genome assembly of *argyrosperma*^14^, which could be partially explained by these SVs. We found that two copies of thaumatin-like protein 1a (*TL1*) and auxin-responsive protein *SAUR32* were disrupted by inversions in the *argyrosperma* genome. We also found a CNV loss spanning the transcription factor *PIF1* and a translocation containing the gene LONG AFTER FAR-RED 3 (*LAF3*). The genomes of *argyrosperma* and *sororia* share some common genes within their unaligned regions, such as microtubule-associated proteins and genes related to tryptophan biosynthesis (Table S2), suggesting that those regions contain highly divergent sequences and are not limited to presence/absence variants. However, other unaligned regions contain more genes in *sororia* than in *argyrosperma*, including the major pollen allergen Ole e 6 (*OLE6*), some proteolytic enzymes and sucrose biosynthetic genes that are absent in the *argyrosperma* genome (Table S2), suggesting that presence/absence variants are also included within the unaligned regions.

### Population data and SNP genotyping

We used samples previously collected throughout Mexico^11^ corresponding to 117 individuals of *argyrosperma*, 50 individuals of *sororia*, 19 feral individuals of *argyrosperma* previously reported to have a semi-wild phenotype and a cultivated genotype based on microsatellite data^11^ and 6 individuals of *C. moschata* (the domesticated sister species of *C. argyrosperma*), that were used as outgroups (Table S3). The samples were sequenced using the tunable Genotype by Sequencing (tGBS) method^20^ to obtain genome-wide genetic information of the *C. argyrosperma* populations. The reads were quality-filtered and mapped against the genome assemblies of *argyrosperma* and *sororia* to predict single nucleotide polymorphisms (SNPs) and assess possible reference biases in the SNP prediction. Using the reference genome of *argyrosperma*, we obtained an initial dataset consisting of 12,813 biallelic SNPs with a mean read depth of 50 reads per SNP and a minor allele frequency (MAF) of at least 1% (13k dataset). We also mapped the whole-genome Illumina reads of *argyrosperma* and *sororia*, as well as the whole-genome sequencing of a *C. moschata* individual and a *C. okeechobeensis* subsp. *martinezii* individual (a closely related wild *Cucurbita* species), against the reference genome of *argyrosperma* to obtain a dataset of 11,498,421 oriented biallelic variants (SNPs and indels) across the genome that was used to assess introgression and incomplete lineage sorting.

### Demographic history of *C. argyrosperma* during its domestication

We eliminated the SNPs that deviated from Hardy–Weinberg equilibrium (exact test with *p* < 0.01) and pruned nearby SNPs under linkage disequilibrium (LD with an *r*^2^ > 0.25 in 100 kbp sliding windows) from the 13k dataset to retrieve a set of 2861 independent SNPs that could be used for demographic analyses.

We found similar genetic variation in *sororia* and *argyrosperma*, regardless of the reference genome used (average nucleotide diversity *π* range 0.095–0.098 for both taxa, see Table S4). At a population scale, the wild population in Jalisco had the highest genetic diversity within *sororia*, while the highest diversity in *argyrosperma* was found in the Pacific Coast of Mexico (Table S5). The domesticated and wild populations of *C. argyrosperma* displayed low genetic differentiation (*F*_ST_ = 0.0646; 95% confidence interval from 0.0565 to 0.0751), while feral populations were more closely related to *argyrosperma* (*F*_ST_ = 0.0479) than with *sororia* (*F*_ST_ = 0.1006).

**Fig. 3:**
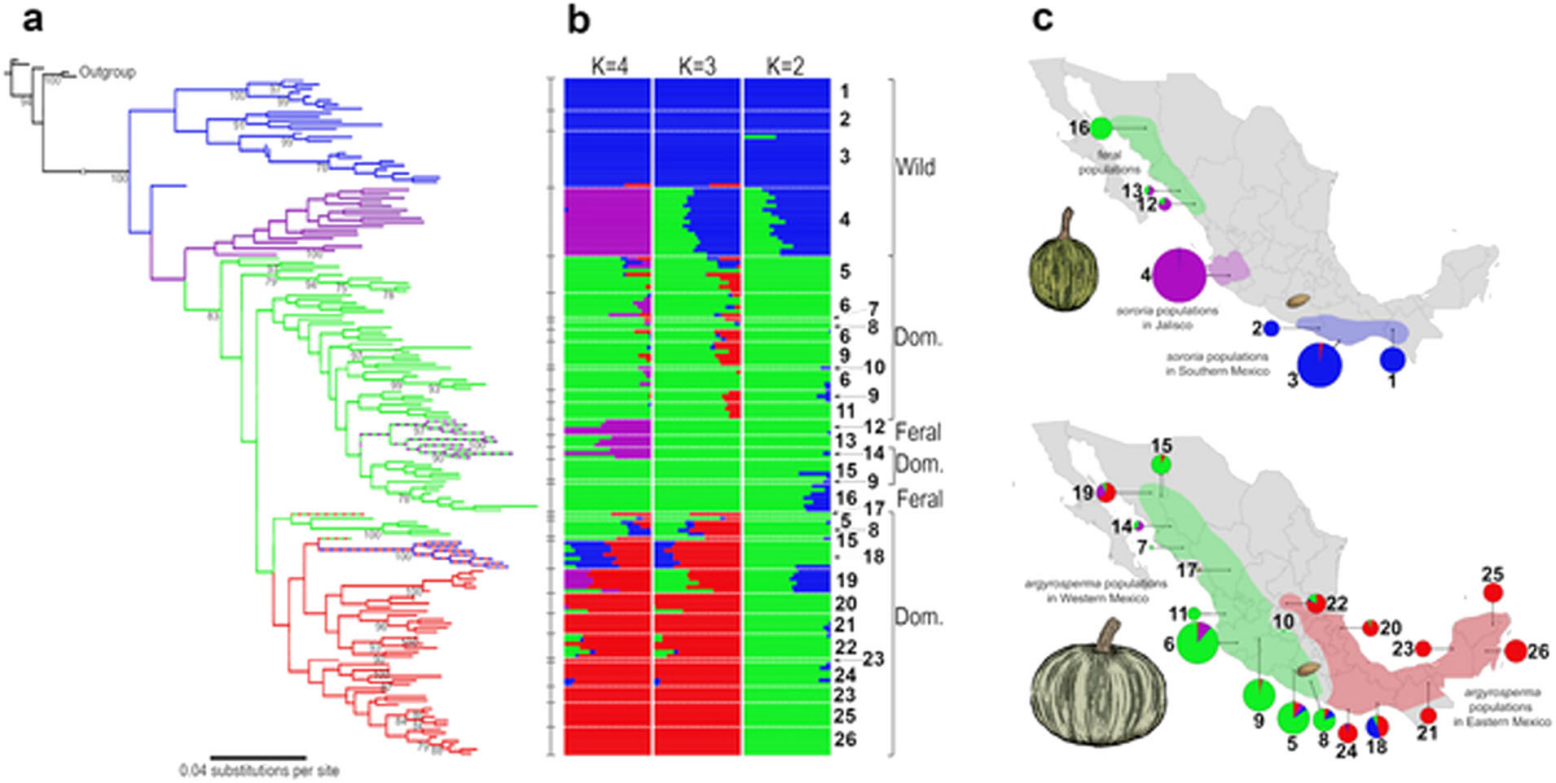
Genetic structure and phylogenetic relationships between the wild and domesticated populations of *Cucurbita argyrosperma* based on 2861 SNPs. **a** Maximum-Likelihood tree with 100 bootstraps (only bootstrap values > 70 shown). **b** ADMIXTURE analysis using *K* values ranging from 2 to 4. **c** Geographic distribution of the wild (top) and domesticated (bottom) populations, with pie chart colors representing the ADMIXTURE assignation of the individuals in *K* = 4 genetic groups (size of pie charts proportional to sample size). The seed in the maps represents the earliest archaeological record of *argyrosperma* from Xihuatoxtla, Guerrero (dated 8700 years BP)^10^

We used SNPhylo^21^ and ADMIXTURE^22^ to evaluate the genealogical relationships and genetic structure among the wild and domesticated populations of *C. argyrosperma*. We confirmed the genetic differentiation between *sororia* and *argyrosperma*, as detected by the *F*_ST_ analyses. Our Maximum-Likelihood (ML) tree groups all the *argyrosperma* populations in a single monophyletic clade (Fig. 3a). We found additional genetic differentiation between the *sororia* populations in Southern Mexico (populations 1–3) and the *sororia* populations in Jalisco (population 4), in both the ADMIXTURE assignations (Fig. 3b) and their positions in the ML tree (Fig. 3a). The *sororia* populations of Jalisco are genetically closer to *argyrosperma*, as shown by their paraphyletic position in the ML tree (Fig. 3a). Consistent with a domestication in the lowlands of Western Mexico, the *argyrosperma* populations of Guerrero and Jalisco represent the basal branches of the *argyrosperma* clade (Fig. 3a), all showing instances of genetic similarity to the *sororia* populations in Jalisco in the four genetic groups (*K*) of ADMIXTURE (Fig. 3b). The *argyrosperma* populations in Western Mexico (populations 5–17) are genetically differentiated from the Eastern populations (populations 18–26), with a possible recent anthropogenic dispersion event of Eastern populations into Onavas, Sonora (population 19; Fig. 3b, c). These four genetic groups are also retrieved in a principal component analysis (PCA; Fig. S2). The ADMIXTURE results (Fig. 3b) uncovered admixture events between some *sororia* and *argyrosperma* populations. This pattern is evident in the populations of Oaxaca and Sinaloa (populations 14 and 18; Fig. 3c). The feral populations are consistently grouped alongside their sympatric domesticated populations within *argyrosperma* (Fig. 3a, b), indicating that these populations diverged recently from nearby domesticated populations. These demographic patterns are robust to different SNP filters, such as different thresholds for missing data, or filtering for significant deviations from Hardy–Weinberg equilibrium (Fig. S3).

**Fig. 4:**
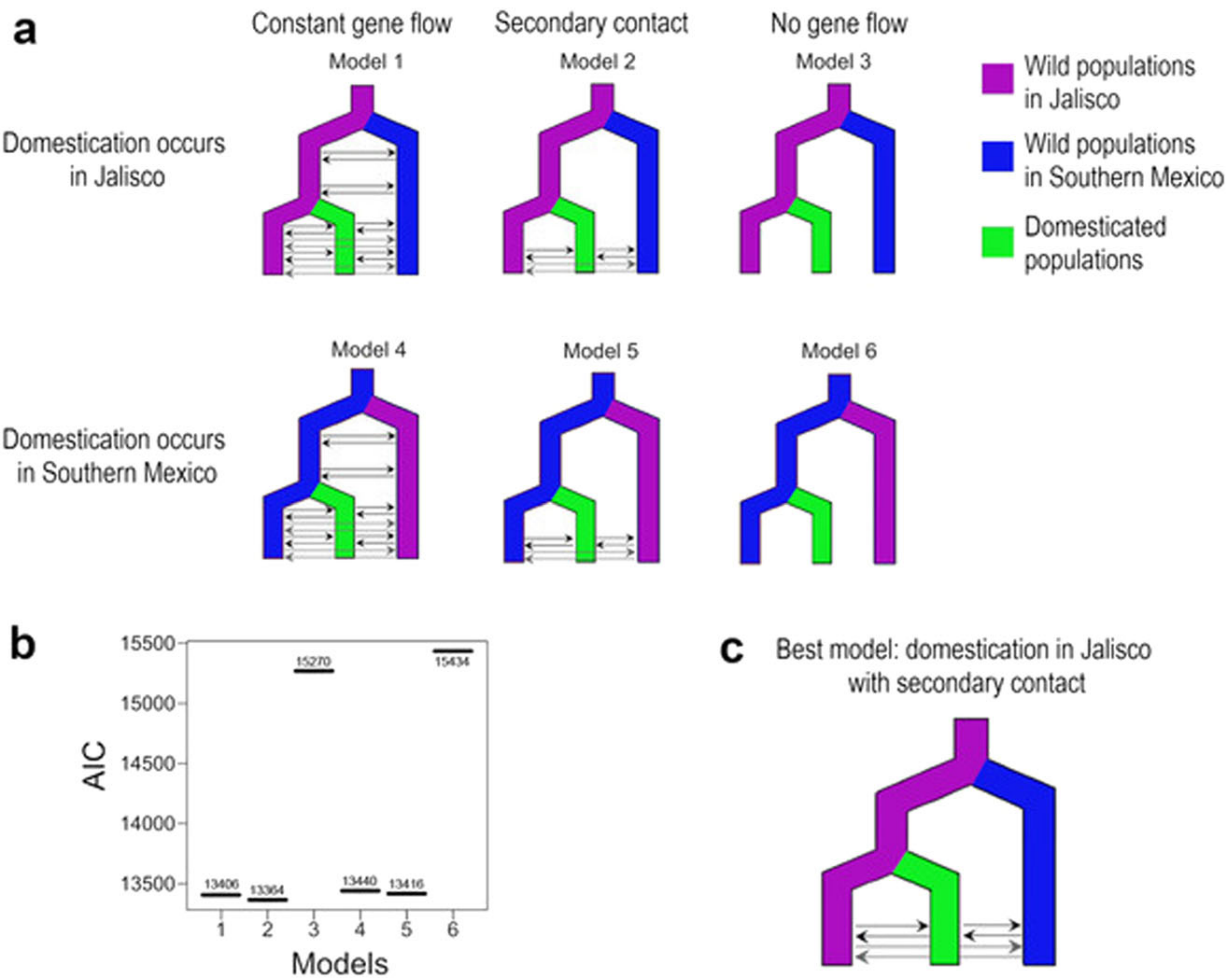
Coalescent simulations and most likely domestication scenario of *Cucurbita argyrosperma*. **a** The six models assessed against the unfolded multidimensional Site Frequency Spectrum of our data. **b** Comparison of the Akaike Information Criterion (AIC) of all the models. **c** The domestication model that best fits the data

We used Fastsimcoal 2^23^ to explicitly test whether *argyrosperma* was domesticated from a *sororia* population in Southern Mexico or from a *sororia* population in Jalisco. Given that gene flow has been previously observed between *argyrosperma* and *sororia*^12^, we compared three different gene flow models (continuous gene flow, secondary contact, or no gene flow) for each scenario (Fig. 4a). A comparison between models using the Akaike Information Criterion indicates that the Jalisco domestication model with secondary contact (*i.e*., extant gene flow after initial genetic isolation between subspecies) is the most likely of the domestication scenarios assayed. We were able to discard the other unrealistic domestication scenarios (i.e., domestication in southern Mexico and lack of gene flow between subspecies), further supporting a domestication event in Jalisco.

### Selection scans in *C. argyrosperma*

**Fig. 5:**
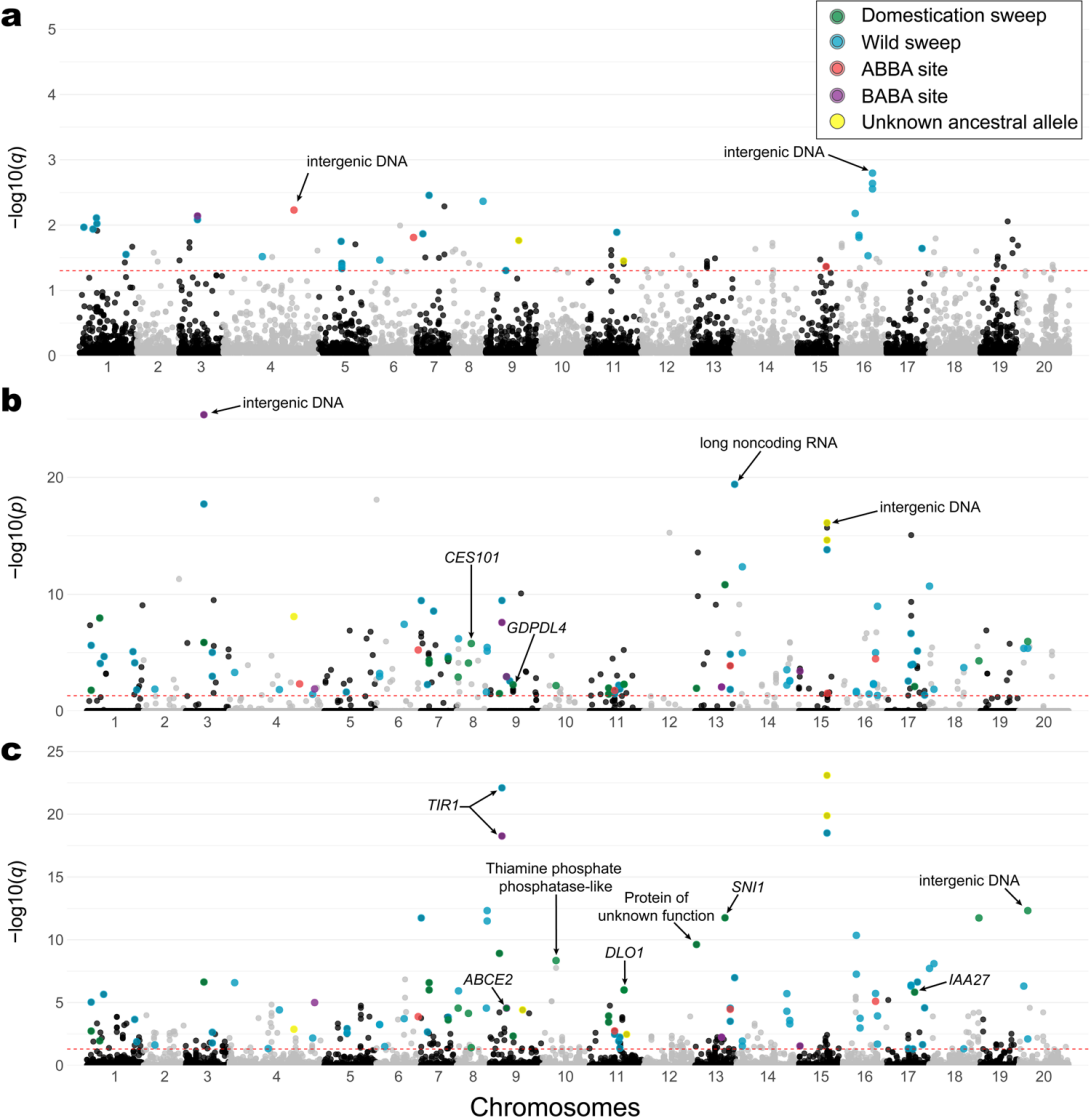
Putative footprints of selection associated with the domestication of *Cucurbita argyrosperma*. Manhattan plots representing the **a** BayeScEnv, **b** PCAdapt, and **c** LFMM 2 tests in each chromosome of the genome. The red dotted line indicates the cutoff value (*q*-value or Bonferroni-corrected *p*-value < 0.05) to determine a candidate locus. Only 110 loci were retrieved as candidate SNPs by more than one test, which are highlighted depending on whether the putative signal of positive selection corresponds to *argyrosperma* (green), *sororia* (blue), an ABBA site (red) a BABA site (purple) or an unknown selective direction (yellow). The arrows indicate the position of some of the discovered candidate genes

In order to perform the tests to detect selective signals associated with the domestication of *C. argyrosperma*, we removed from the 13k dataset the *C. moschata* individuals as well as the feral individuals of *C. argyrosperma*. We used the 1% MAF threshold for this subset, obtaining a 10,617 SNP dataset suitable to detect selective signals, with a marker density of 44 SNPs per Mb. LD was limited within the dataset, with a mean pairwise *r*^2^ of 0.1, and with *argyrosperma* showing a faster LD decay than *sororia* (Fig. S4). We performed three outlier tests as implemented in BayeScEnv^24^, PCAdapt^25^, and LFMM 2^26,27^ to detect selective signals between the domesticated and the wild populations of *C. argyrosperma* (Fig. 5). BayeScEnv is an *F*_ST_-based method that tests correlation with other variables, in our case the wild or domesticated nature of each population (coded as 0 and 1, respectively). PCAdapt does not require an a priori grouping of individuals into wild/domesticated, as we used the two principal components of a PCA to control for the underlying genetic structure between subspecies (Fig. S2). LFMM 2 identifies the number of latent factors in the populations through least square estimates to find correlations between genetic variants and the domesticated or wild phenotypes in the dataset^26^. In order to reduce the number of false positives, we only analyzed the SNPs that were detected as outliers by at least two of the tests.

We discovered 110 SNPs that converged as candidates in at least two tests (Fig. 5). We used *C. moschata* and *C. okeechobeensis* subsp. *martinezii* as outgroups to determine the direction of the putative selective pressures for each candidate SNP, as well as determining possible introgression or incomplete lineage sorting between *sororia*, *argyrosperma* and *C. moschata*. We found that 22 of the candidate SNPs corresponded to selective signals in *argyrosperma*, while 70 candidate SNPs corresponded to selective signals in *sororia* (Fig. 5). We could not determine the direction of selection for 5 candidate SNPs, and 13 showed signals of introgression or incomplete lineage sorting.

We identified several instances of either genetic introgression or incomplete lineage sorting between *C. moschata* and both *argyrosperma* (ABBA sites) or *sororia* (BABA sites) (Fig. S5). We performed a genome-wide *D*-statistic analysis and found significantly more instances of shared derived variants between *argyrosperma* and *C. moschata* than between *sororia* and *C. moschata* (block-jackknife *p-*value = 0.0014), obtaining an overall admixture fraction *f*_G_ of 0.01 (Table S6). From the 13 candidate SNPs with signals of introgression, 6 correspond to ABBA sites and 7 correspond to BABA sites (Fig. 5).

We found 45 protein-coding genes and one long-noncoding RNA including candidate SNPs within their structure (i.e., introns, exons, UTRs), which were assigned according to the observed direction of the putative selective signals (Table S7). Among the genes under putative selection in *sororia* were four serine/threonine-protein kinases, including *PBL10* and *PBL23* (Table S7). Among the genes under putative selection in *argyrosperma*, we found glycerophosphodiester phosphodiesterase *GDPDL4*, auxin-responsive protein *IAA27*, ABC transporter E family member 2 (*ABCE2*), *DMR6*-like oxygenase 1 (*DLO1*), and serine/threonine-protein kinase *CES101* (Table S7). We also found several genes under putative selection overlapping ABBA and BABA sites (Table S7). We found a transport inhibitor response 1 (*TIR1*) homolog under putative selection in both *sororia* and as a BABA site. Curiously, the gene *MKP1* was found under selection in both *argyrosperma* and in *sororia*.

## Discussion

The genome assembly of *sororia* represents the first high-quality sequenced genome of a wild *Cucurbita*, which allowed us to detect structural and functional differences with the *argyrosperma* genome. The genome assembly of *argyrosperma* was smaller than the *sororia* assembly, which is possibly caused by the loss of structural variants during its domestication, as has been reported in pan-genome studies^28^. Many of these unaligned regions contain entire genes in *sororia*, making this wild taxon a reservoir of potentially adaptive presence/absence variants. However, the extant genetic diversity of *argyrosperma* is similar to that of *sororia*, which suggests that the effects of the domestication bottleneck were alleviated by the current gene flow between both subspecies as suggested by our coalescent simulations and by the results of the previous studies^12^. The fast LD decay in *argyrosperma* further supports the limited effect of the domestication bottleneck. This gene flow may be related to the sympatric distribution of the wild and domesticated populations of *C. argyrosperma* throughout the Pacific Coast of Mexico^11^, where their coevolved pollinator bees *Peponapsis* spp. and *Xenoglossa* spp. are found^29^. Traditional agricultural practices are another fundamental force that maintains the diversity of crop species^30^. Since *argyrosperma* is a traditional crop cultivated for both self-supply and local markets where it has a specialized gastronomic niche^13^, the genetic diversity in *argyrosperma* is also maintained by the conservation of local landrace varieties at local scales^31,32^.

**Fig. 6:**
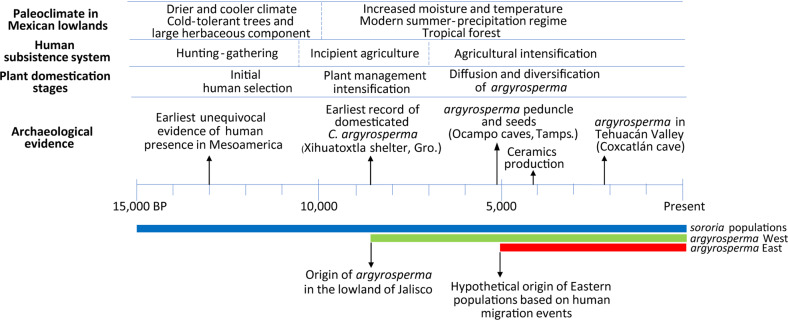
Model of *Cucurbita argyrosperma* domestication based on archaeological^6,33–35^ and genetic data. The colored lines represent the presence of each *C. argyrosperma* genetic cluster in Mesoamerica (blue = *sororia* populations, green = *argyrosperma* populations in Western Mexico, red = *argyrosperma* populations in Eastern Mexico)

Our demographic analyses suggest that the extant populations from Jalisco are the closest modern relatives of the initial population of *sororia* from which *argyrosperma* originated. The genetic relatedness between the *sororia* populations from Western Mexico and *argyrosperma* was also observed with mitochondrial markers^6^. The domestication of *C. argyrosperma* likely started around 8,700 years ago, as suggested by the earliest, albeit taxonomically ambiguous, archaeological record of *argyrosperma*^10,33^. Since crop domestication in Mesoamerica is linked to migration patterns and cultural development of early human populations in America^7,34^, we expected *argyrosperma* to share historical demography patterns with human history. Our data shows that the *argyrosperma* populations found in Guerrero and Jalisco are the most closely related to the *sororia* populations from Jalisco. This means that even if the closest wild relatives of *argyrosperma* are currently found in Jalisco, the domestication process may have occurred throughout the lowlands of Jalisco and the Balsas basin^10^. Ancient human migration events have been proposed to occur along the river basins in Southwestern Mexico, which may explain the genetic cohesiveness among the *argyrosperma* populations of that area that represent the first fully domesticated lineage of the species^34^. Previous studies based on 8,700 years old phytoliths found in Xihuatoxtla, Guerrero, suggest the co-occurrence of *Zea mays* and *C. argyrosperma* in the Balsas region within this time period^10,33^. Overall, the patterns of genetic structure for *C. argyrosperma* are coherent with the archaeological evidence of early human migration throughout Mesoamerica^35,36^ (Fig. 6).

We found several signals of putative selection between *argyrosperma* and *sororia*, even though tGBS sequencing has a limited capacity to detect selective signals across the genome^37^. The SNP density for our selection tests was of 44 SNPs per Mb, which is one order of magnitude denser than other studies using reduced-representation genome sequencing to detect selective signals^37^. This is a consequence of the relatively small genome size of *C. argyrosperma*^14^. Nonetheless, the LD in *C. argyrosperma* decays at a shorter length than our SNP density, so several signals of selection might be missing from our scans, and some signals might not correspond to the actual gene under selection, but rather to a neighboring region in the genome^37^. Therefore, our genome scans should be interpreted as a partial representation of the selective signals associated with the domestication process^37^.

Most of the putative selective signals were attributed to *sororia*, probably because wild taxa are subject to many natural selective pressures. Many of the SNPs that were retrieved as outliers were found on genes involved in biotic and abiotic plant defense responses. For example, *PBL10* and *PBL23* have been suggested to be involved in plant defense pathways due to their similarity to other serine/threonine-protein kinases^38^.

However, we also found some genes under putative selection in *argyrosperma* that could be attributed to defense mechanisms such as *DLO1*, which is involved in pathogenic defense responses^39^. We found *GDPDL4* under putative selection, which is involved in trichome differentiation^40^, a morphological characteristic that differentiates *argyrosperma* from *sororia* (Fig. 1). We also found two disrupted copies of *TL1* and the absence of *OLE6* within the *argyrosperma* genome, both of which cause allergic reactions in humans^41,42^. This is concordant with previous studies showing that selective pressures during domestication actively purge these defense mechanisms, as the products of these responses are usually unpleasant or harmful to humans when the plant is consumed^43^. This is particularly important for breeding programs, since wild *Cucurbita* such as *sororia* harbor loci associated with disease resistance that their domesticated counterparts have lost^4^. The selective pressures found in *MKP1* suggest a disruptive selection regime between *sororia* and *argyrosperma*. Since *MKP1* modulates defense responses^44^, it is possible that both subspecies adapted to differential environmental pressures as domestication took place.

We found several candidate genes involved in the regulation of ABA. The alteration of growth hormones may play an important role in *C. argyrosperma* domestication. ABA is involved in a myriad of functions, such as the regulation of plant growth, plant development, seed dormancy, and response to biotic/abiotic stress^45^. In this sense, the lack of dormancy in seeds and gigantism are both common domestication changes that are present in domesticated cucurbits that may be caused by changes in the regulation of ABA and brassinosteroids^8,46^. We found *SAUR32*, *PIF1*, and *LAF3* within the high-confidence SVs in *argyrosperma*, all of which act as inhibitors of seed germination under dark conditions^47–49^ and may explain the lack of seed dormancy in *argyrosperma*. Likewise, we found *IAA27* under selection in *argyrosperma*, which is involved in plant growth and development^50^.

Selection over seed size has been particularly important in the domestication of *C. argyrosperma*^9^. We found *ABCE2* under selection in *argyrosperma*. Some ABC transporters are involved in the transmembrane transport of ABA-GE, an ABA conjugate that is usually attributed to the plant response against water stress^51^. However, previous studies in *Hordeum vulgare* suggest that the transport of ABA-GE may play a role in seed development alongside *de novo* ABA synthesis within the developing seed^52^, suggesting a role of ABC transporters in the seed development of *C. argyrosperma*. Previous studies have also identified an association between variants in ABC transporter proteins and seed size in *Cucurbita maxima*^53^ and *Linum usitatissimum*^54^, further suggesting that ABA may be deregulated via selective pressures on ABC transporters to enhance seed size in *C. argyrosperma* during its domestication. Variants in a serine/threonine-protein kinase, as the one we found in our selective scans, have also been associated with seed size in *C. maxima*^53^.

We found shared derived variants between *C. moschata* and *C. argyrosperma* under putative selection. This suggests that, given the close relationship between *C. argyrosperma* and *C. moschata*, both species may share domestication loci involved in common domestication traits. However, we also found *TIR1* under selection in *sororia* and as a BABA site, which is an auxin receptor involved in ethylene signaling and antibacterial resistance in roots^55^. The introgression of wild alleles into domesticated *Cucurbita* has been previously reported as an effective method to improve the resistance of domesticated crops^56^. Along this line, our results suggest that *sororia* is an important source of adaptive alleles for *C. moschata*. ABBA and BABA sites under selection may be shared with *C. moschata* either due to incomplete lineage sorting or by adaptive introgression with the wild and domesticated populations of *C. argyrosperma*. The incorporation of domesticated loci between *C. argyrosperma* and *C. moschata* through introgression may have been an effective way for Mesoamerican cultures to domesticate multiple *Cucurbita* taxa. This hypothesis is supported by the significant amount of ABBA sites shared between the genomes of *C. argyrosperma* and *C. moschata*. However, this hypothesis needs to be further addressed using population-level data of *C. moschata* with other wild and domesticated *Cucurbita* species.

## Materials and methods

### Genome assembly and annotation of *Cucurbita argyrosperma* subsp. *sororia*

We sequenced and assembled *de novo* the genome of a *sororia* individual collected in Puerto Escondido (Oaxaca, Mexico). Its DNA was extracted from leaf tissue and sequenced using PacBio Sequel at the University of Washington PacBio Sequencing Services and using Illumina HiSeq4000 at the Vincent J. Coates Genomics Sequencing Laboratory in UC Berkeley (NIH S10 Instrumentation Grants S10RR029668 and S10RR027303). We filtered the Illumina sequences using the qualityControl.py script (https://github.com/Czh3/NGSTools) to retain the reads with a PHRED quality ≥ 30 in 85% of the sequence and an average PHRED quality ≥ 25. The Illumina adapters were removed using SeqPrep (https://github.com/jstjohn/SeqPrep) and the paired reads that showed overlap were merged. The chloroplast genome was assembled using NOVOplasty^57^ and the organellar reads were filtered using Hisat2^58^ against the chloroplast genome of *argyrosperma*^14^ and the mitochondrial genome of *C. pepo*^59^. We assembled the nuclear genome into small contigs using the Illumina reads and the Platanus assembler^60^. The Platanus contigs were assembled into larger contigs using the PacBio Sequel reads and DBG2OLC^61^. We performed two iterations of minimap2 and racon^62^ to obtain a consensus genome assembly by mapping the PacBio reads and the Platanus contigs against the DBG2OLC backbone. We performed three additional polishing steps using PILON^63^ by mapping the Illumina reads against the consensus genome assembly with BWA *mem*^64^.

The genome annotation processes were performed using the GenSAS v6.0 online platform^65^. The transposable elements within the genome were predicted and masked using RepeatModeler (http://www.repeatmasker.org/RepeatModeler/). We downloaded five RNA-seq libraries of *C. argyrosperma* available on the Sequence Read Archive (accessions SRR7685400, SRR7685404–SRR7685407) to use them as RNA-seq evidence for the gene prediction. We performed the same quality filters described above for the RNA-seq data and aligned the high-quality reads against the masked genome of *C. argyrosperma* subsp. *sororia* using STAR v2.7^66^. We used filterBAM from the Augustus repository^67^ to filter low-quality alignments and used the remaining alignments as RNA-seq evidence to predict the gene models using BRAKER2^17^. The gene predictions were functionally annotated using InterProScan^68^ and by aligning the gene models against the SwissProt database^69^ using BLASTp^70^ with an *e*-value < 1e^−6^. We performed a manual assessment of the predicted gene models to eliminate annotation artifacts.

### Anchoring the reference genomes into pseudomolecules

We aimed to improve the genome assemblies of *argyrosperma* and *sororia*, which were assembled in 920 scaffolds^14^ and 817 contigs, respectively. Thus, we generated PacBio corrected reads from the published PacBio RSII reads of *argyrosperma* (NCBI SRA accession SRR7685401) and the PacBio Sequel reads of *sororia* (sequenced for this study at the University of Washington PacBio Sequencing Services) using CANU^71^. The macrosynteny of *Cucurbita* genomes is largely conserved between species^72^, so we performed a reference-guided scaffolding step using RaGOO^18^ to anchor the genome assemblies of *argyrosperma*^14^ and *sororia* into pseudomolecules. We used the genome assembly of *C. moschata*^16^ as the reference genome for RaGOO^18^ and we used the PacBio corrected reads of each taxon to detect and correct misassemblies, using a gap size of 2600 bp for chromosome padding (i.e., we filled the gaps between contigs with 2600 stretches of Ns, corresponding to the average gap length of the *argyrosperma* genome assembly). The chromosome numbers in both assemblies were assigned in correspondence to the genome assembly of *C. moschata*^16^.

### Structural variant analysis

We evaluated the synteny between *Cucurbita* genomes using Synmap2^73^. We predicted the SVs between *argyrosperma* and *sororia* using SyRI^74^ alongside nucmer^75^ with a minimum cluster length of 500 bp, an alignment extension length of 500 bp, a minimum match length of 100 bp and a minimum alignment identity of 90%. We also used Sniffles alongside NGMLR^76^ as an additional SV predictor, by aligning the *argyrosperma* PacBio reads against the genome assembly of *sororia* (only the SVs with a minimum support of 6 reads were retained). We only analyzed the SVs that were independently predicted by SyRI and Sniffles. Only the SVs that overlapped within a range of ±100 bp at the start and end positions of each prediction were retained as high-confidence SVs. Due to the limitations of read-mapping techniques to predict presence–absence variants, we only analyzed the unaligned regions predicted by SyRI for this type of variants.

The gene content associated with each type of structural variant was considered either as the overlap between genes and variants (inversions and translocations) or as the genes contained entirely within the structural variants (CNVs and unaligned regions). We performed a Gene Ontology enrichment analysis using topGO and the *weight01* algorithm^77^ to find enriched biological functions associated with each type of structural variant. We determined the significantly enriched biological functions by performing Fisher’s exact test (*p*-value < 0.05). We plotted the genome rearrangements and SVs between *sororia* and *argyrosperma* using Smash^78^ with a minimum block size of 100,000 bp, a threshold of 1.9, and a context of 28.

### Data filtering and SNP genotyping

We used previously collected seeds from 19 populations of *argyrosperma* landraces, four populations of *sororia*, and three feral populations^11^, covering most of the reported distribution of this species throughout Mexico^5^ (Table S3). Each of the collected seeds came from a different maternal plant, in order to avoid signals of inbreeding. The seeds were germinated in a greenhouse and total DNA was extracted from fresh leaves using a DNeasy Plant MiniKit (Qiagen) of 192 individuals across the collected populations (Table S3), including five individuals of *C. moschata* to be used as outgroup. All 192 individuals were sequenced by Data2Bio LLC using the tunable Genotyping by Sequencing (tGBS) method^20^ with an Ion Proton instrument and two restriction enzymes (Sau3AI/BfuCI and NspI). The wild and domesticated populations were randomly assigned to the plate wells before library preparation to avoid sequencing biases.

The raw reads of the tGBS sequencing were trimmed using LUCY2^79^, removing bases with PHRED quality scores < 15 using overlapping sliding windows of 10 bp. Trimmed reads shorter than 30 bp were discarded. The trimmed reads were mapped against the genome assembly of *argyrosperma* using segemehl^80^, since empirical studies suggest this read-mapping software outperforms others for Ion Torrent reads^81^. We only retained the reads that mapped uniquely to one site of the reference genome for subsequent analyses.

We used BCFtools^82^ for an initial variant calling step, retaining variants with at least 6 mapped reads per individual per site where the reads had a minimum PHRED quality score of 20 in the called base and a minimum mapping quality score of 20^83^. We used plink^84^ to perform additional filters, such as retaining only biallelic SNPs, retaining SNPs with no more than 50% of missing data, individuals with no more than 50% of missing data and sites with a minor allele frequency (MAF) of at least 1% (13k dataset). After eliminating individuals with missing data, 109 individuals of *argyrosperma*, 44 individuals of *sororia*, 14 feral individuals and 5 individuals of *C. moschata* remained for the subsequent analyses. We repeated the SNP prediction using the reference genome of *sororia* to evaluate potential reference biases. We found a similar number of SNPs (10,990) and comparable estimates of genetic diversity (see Table S4), suggesting that reference bias does not have a meaningful impact on our results. Thus, we employed the domesticated genome as the reference for the rest of the population analyses. We repeated our analyses using a separate filtering step of missing data for the domesticated and the wild populations, retrieving 84.18% of the SNPs from the 13k dataset and obtaining the same results (Fig. S3).

In order to obtain an adequate SNP dataset to infer the demographic history of *C. argyrosperma*, we performed additional filters to the 13k dataset with plink^84^, including (i) the elimination of all the SNPs that diverged significantly (*p* < 0.01) from the Hardy–Weinberg equilibrium exact test^85^ to remove potential allelic dropouts, (ii) the elimination of adjacent SNPs with a squared correlation coefficient (*r*^2^) larger than 0.25 within 100 kbp sliding windows with a step size of 100 bp. We repeated the demographic analyses without filtering the SNPs with significant deviations from Hardy–Weinberg equilibrium, obtaining the same results (Fig. S3).

We also generated a SNP dataset to detect selective signals associated with the domestication of *C. argyrosperma* by eliminating all the feral individuals of *C. argyrosperma*, which could not be assigned to either a wild or a domesticated population, as well as the five individuals of *C. moschata*. We also eliminated the SNP sites with more than 50% missing data and performed a MAF filter of 1% after reducing the number of individuals in the 13k dataset. The SNP density was calculated with VCFtools^86^ and the LD decay was calculated using plink^84^ with a minimum *r*^2^ threshold of 0.001.

We also sequenced the genome of a *C. moschata* individual from Chiapas (Mexico) and the genome of a *C. okeechobeensis* subsp. *martinezii* individual from Coatepec (Veracruz, Mexico) using the Illumina HiSeq4000 platform in UC Berkeley, to evaluate possible introgression and incomplete lineage sorting with *C. argyrosperma*. We downloaded the genome sequences of *argyrosperma*^14^ from the Sequence Read Archive (accessions SRR7685402 and SRR7685403). The Illumina whole-genome sequences were filtered using the same quality parameters as the ones used in the genome assembly of *sororia* (see above) and were aligned against the genome assembly of *argyrosperma* using BWA *mem*^64^. We only retained the reads that mapped uniquely to one site of the reference genome and retained only the biallelic sites with a sequencing depth > =10 reads per genome.

### Population structure

We used diveRsity^87^ to calculate the pairwise *F*_ST_ statistics, using 100 bootstraps to calculate the 95% confidence intervals. We estimated the genetic variation in the wild, domesticated and feral populations with STACKS^88^. Using ADMIXTURE^22^, we evaluated the genetic structure among the *sororia* and *argyrosperma* populations, evaluating their individual assignment into one (CV error = 0.26205), two (CV error = 0.25587), three (CV error = 0.25806) and four (CV error = 0.26658) genetic groups (*K*). We reconstructed a maximum-likelihood tree with SNPhylo^21^, based on substitutions per site between all the individuals with 100 bootstraps to assess the reliability of the tree topology. We used plink^84^ to perform a principal component analysis (PCA) using 10 principal components.

### Coalescent simulations

We used Fastsimcoal 2^23^ to determine the parameters that maximize the composite likelihood of each model given the unfolded multidimensional SFS. The unfolded multidimensional SFS was obtained with DADI^89^, using 17 *sororia* individuals of Jalisco, 27 *sororia* individuals of Southern Mexico, 109 *argyrosperma* individuals and 5 *C. moschata* individuals as an outgroup to unfold the SFS. We ran 100,000 simulations with 20 replicates for each model (two divergence scenarios and three gene flow scenarios) using the following settings: a parameter estimation by Maximum Likelihood with a stopping criterion of 0.001 difference between runs, a minimum SFS count of 1, a maximum of 40 loops to estimate the SFS parameters and a maximum of 200,000 simulations to estimate the SFS parameters. We also selected log-uniform priors for parameter estimations, setting times of divergence between 1000 and 200,000 generations (domestication times are expected to fall within this interval, given that the presumed most ancient evidence of the human presence in America is 33,000 years old^90^; while the split between the wild populations is expected to coincide with the extinction of the megafauna in America, which acted as the natural dispersers of wild *Cucurbita* during the Pleistocene^3^), effective population sizes (*N*_e_) between 100 and 60,000 individuals and migration rates (m) between 0.0001 and 0.5. The constant gene flow scenarios calculated a migration rate matrix throughout the simulation, from the present back to the common ancestral population of all lineages, with independent migration rates for each possible direction of gene flow. The secondary contact scenarios simulated a migration matrix only at the start of the simulation, before the coalescence event between the wild and domesticated lineages (see Data S1–S6 for detailed model parameters). We also constrained the times of divergence in all scenarios by forcing the domesticated taxa to diverge after the wild one. Each generation in the model corresponds to one calendar year, as this species displays an annual life cycle^13^. The 20 replicates of each model converged to similar likelihoods, indicating that the simulations performed well. After corroborating that all replicates converged to similar likelihoods, we combined all replicates and retained all outputs that were above 95% of the likelihood distribution. We found that the Jalisco model of divergence with secondary contact had the lowest Akaike Information Criterion values for all the tested models.

### Tests to detect selective signals and introgression

We used BayeScEnv^24^ to detect putative regions under selection that were differentiated between *sororia* and *argyrosperma*. For the “environmental” values used by BayeScEnv, we assigned each population as either wild (0) or domesticated (1). We ran two independent MCMC analyses with 20 initial pilot runs with a length of 10,000 generations and a main run with an initial burn-in of 100,000 generations and a subsequent sampling step for 100,000 generations sampling every 20 generations. We confirmed the convergence between both chains using the Gelman and Rubin statistic^91^. The SNPs with *q-*values < 0.05 were regarded as candidate loci under selection.

The Mahalanobis distances implemented in PCAdapt^25^ were used to detect candidate SNPs after controlling for the first two principal components in our dataset, which correspond to the subspecies and geographical differentiation observed among the populations (see Fig. S2). We performed Bonferroni corrections to adjust the *p-*values obtained from PCAdapt and the SNPs with Bonferroni-corrected *p-*values < 0.05 were regarded as candidate loci under selection.

We used LFMM 2^26^ to identify candidate SNPs differentiating the wild and domesticated phenotypes of *C. argyrosperma*. We tested *K* number of latent factors from 1 to 10 using sNMF^27^ and determined an optimal *K* = 6. We used 6 latent factors and a ridge penalty to identify significant associations between the response (wild or domesticated phenotypes) and the genetic variants. Finally, we performed FDR corrections to obtain *q-*values, with *q*-values < 0.05 being regarded as candidate loci under selection.

We performed an ABBA-BABA test using Dsuit^92^ against the 11,498,421 whole-genome variants to evaluate signals of introgression or incomplete lineage sorting between *argyrosperma*, *sororia* and *C. moschata*, while using *C. okeechobeensis* subsp. *martinezii* as an outgroup. We calculated a global *D*-statistic by performing a SNP-by-SNP analysis to determine the amount of ABBA and BABA sites throughout the entire genome. We also calculated local *D*-statistics within 500 SNP windows with a step size of 250 SNPs. We used the five tGBS data of *C. moschata*, as well as the whole-genome sequences of *C. moschata* and *C. okeechobeensis* subsp. *martinezii*, to define the ancestral state of each candidate locus from the selection scans and determine the direction of the selective signals or whether they corresponded to ABBA or BABA sites. We used snpEff^93^ to associate the candidate loci found in at least two tests with the genome annotation of *argyrosperma*^14^.

## Supplementary information

Supplementary information

## Data Availability

All the raw sequencing data and genome assemblies are available in the National Center of Biotechnology Information under the BioProject accession PRJNA485527 (genome accesions SDJN00000000 and JAGKQH010000000; SRA accesions available in Table S3). The genome assemblies are also available in Figshare (https://doi.org/10.6084/m9.figshare.14370584.v1) and at the Cucurbit Genomics Database^94^.
