# Supplementary material for: The domestication of Cucurbita argyrosperma as revealed by the genome of its wild relative
Source: Hortic Res. 2021 May 1;8:109. doi: 10.1038/s41438-021-00544-9 (PMC8087764; doi:10.1038/s41438-021-00544-9)
Supplement: Supplementary file 1 — Supplementary information [file 41438_2021_544_MOESM1_ESM.docx]

Supplementary information for:

**The domestication of *Cucurbita argyrosperma* as revealed by the genome of its wild relative**

Josué Barrera-Redondo, Guillermo Sánchez-de la Vega, Jonás A. Aguirre-Liguori, Gabriela Castellanos-Morales, Yocelyn T. Gutiérrez-Guerrero, Xitlali Aguirre-Dugua, Erika Aguirre-Planter, Maud I. Tenaillon, Rafael Lira-Saade, Luis E. Eguiarte

Contact: [josue_barrera@comunidad.unam.mx](mailto:josue_barrera@comunidad.unam.mx), [rlira@unam.mx](mailto:rlira@unam.mx) and [fruns@unam.mx](mailto:fruns@unam.mx)

**This file includes:**

**Supplementary Tables**

S1: Assembly metrics of the *argyrosperma* and *sororia* genomes.

S2: Putative rearrangements between *argyrosperma* and *sororia.*

S3: Information of 192 individuals sequenced with tGBS.

S4: Genetic diversity of *C. argyrosperma* subspecies.

S5: Genetic diversity of each *C. argyrosperma* population.

S6: Introgression analysis using the ABBA-BABA test.

S7: Candidate genes under selection.

**Supplementary Figures**

S1: Gene synteny dot plots between *Cucurbita* genomes.

S2: Principal Component Analyses.

S3: Demographic analyses using alternative SNP filters.

S4: LD decay in *C. argyrosperma*.

S5: ABBA-BABA test using sliding windows.

**Supplementary Data**

S1: Coalescent model 1 (domestication in Jalisco with constant gene flow)

S2: Coalescent model 2 (domestication in Jalisco with secondary contact)

S3: Coalescent model 3 (domestication in Jalisco with no gene flow)

S4: Coalescent model 4 (domestication in southern Mexico with constant gene flow)

S5: Coalescent model 5 (domestication in southern Mexico with secondary contact)

S6: Coalescent model 6 (domestication in southern Mexico with no gene flow)

**Supplementary Tables**

**Table S1.** **Assembly metrics of the genome of *Cucurbita argyrosperma* subsp. *sororia* and *C. argyrosperma* subsp. *argyrosperma*, before and after RaGOO scaffolding.**

| **Metrics** | ***C. argyrosperma* subsp. *sororia*** | | ***C. argyrosperma* subsp. *argyrosperma*** | |
| --- | --- | --- | --- | --- |
|  | **Before RaGOO** | **After RaGOO** | **Before RaGOO** | **After RaGOO** |
| Assembly size (bp) | 252,760,152 | 255,194,784 | 228,814,150 | 231,583,150 |
| No. of contigs | 817 | 959 | 1,481 | 1,653 |
| No. of scaffolds | 817 | 72 | 920 | 27 |
| Longest contig (bp) | 4,976,248 | 4,922,130 | 2,172,140 | 2,172,140 |
| Longest scaffold (bp) | 4,976,248 | 21,187,142 | 2,746,581 | 22,228,640 |
| Contig N50 (bp) | 1,323,288 | 1,205,533 | 463,388 | 447,042 |
| Scaffold N50 (bp) | 1,323,288 | 12,094,557 | 620,880 | 11,677,370 |
| Contig L50 | 58 contigs | 60 contigs | 132 contigs | 134 contigs |
| Scaffold L50 | 58 scaffolds | 9 scaffolds | 103 scaffolds | 9 scaffolds |
| No. of contigs > 1 kb | 817 (100.0%) | 959 (100.0%) | 1,481 (100.0%) | 1,625 (98.3%) |
| No. of contigs > 10 kb | 799 (97.8%) | 941 (98.1%) | 1,417 (95.7%) | 1,527 (92.4%) |
| No. of contigs > 100 kb | 289 (35.4%) | 325 (33.9%) | 493 (33.3%) | 492 (29.8%) |
| No. of scaffolds > 10kb | 799 (97.8%) | 62 (86.1%) | 903 (98.2%) | 25 (92.6%) |
| No. of scaffolds > 100kb | 289 (35.4%) | 25 (34.7%) | 455 (49.5%) | 20 (74.1%) |
| No. of scaffolds > 1Mb | 81 (9.9%) | 20 (27.8%) | 51 (5.5%) | 20 (74.1%) |
| CG content | 36.54%  213x  75.4x  30,592  3,318  92.8%  1.2%  6.0% | | 36.45%  120x  31x  27,998  3,476  93.2%  0.9%  5.9% | |
| Illumina read coverage |  |  |  |  |
| PacBio read coverage |  |  |  |  |
| No. of genes |  |  |  |  |
| Average gene size (bp) |  |  |  |  |
| Complete BUSCOs |  |  |  |  |
| Fragmented BUSCOs |  |  |  |  |
| Missing BUSCOs |  |  |  |  |

**Table S2.** **High-confidence structural variants found between the genomes of *C. argyrosperma* subsp. *sororia* (wild genome) and *C. argyrosperma* subsp. *argyrosperma* (domesticated genome), as predicted by both SyRI and Sniffles.**

| **Structural variants** | **Number of variants** | **Cumulative size of variants (bp)** | **Genes within variants** | **Enriched GO biological functions (*p*-value)** |
| --- | --- | --- | --- | --- |
| * Copy-gain variants | 159 | 162,758 | 3 | DNA replication (0.0088) |
| * Copy-loss variants | 259 | 454,056 | 12 | Proton transmembrane transport (5.5 e^-5^) |
| Translocations | 22 | 73,002 | 23 | DNA topological change (0.0065) |
|  |  |  |  | Fatty acid biosynthetic process (0.0409) |
| Inversions | 3 | 19,271 | 4 | DNA replication (0.017) |
|  |  |  |  | Response to auxin (0.025) |
| ** Unaligned regions in domesticated genome | 2479 | 18,979,604 | 149 | Cytoskeleton organization (0.0045) |
|  |  |  |  | NADP biosynthetic process (0.0182) |
|  |  |  |  | Tryptophan biosynthetic process (0.0212) |
|  |  |  |  | Negative regulation of translation (0.0272) |
|  |  |  |  | Protein ubiquitination (0.0275) |
|  |  |  |  | Lysine biosynthetic process via diaminopimelate (0.0302) |
|  |  |  |  | Inositol phosphate dephosphorylation (0.0391) |
|  |  |  |  | Translational termination (0.0450) |
| ** Unaligned regions in wild genome | 3846 | 28,437,839 | 637 | Proteolysis (0.0005) |
|  |  |  |  | Tryptophan biosynthetic process (0.0012) |
|  |  |  |  | DNA replication initiation (0.0050) |
|  |  |  |  | Phosphorelay signal transduction system (0.0123) |
|  |  |  |  | ATP synthesis coupled proton transport (0.0130) |
|  |  |  |  | Negative regulation of DNA helicase activity (0.0134) |
|  |  |  |  | Glucosylceramide catabolic process (0.0266) |
|  |  |  |  | Sucrose biosynthetic process (0.0266) |
|  |  |  |  | Cytoskeleton organization (0.0307) |
|  |  |  |  | L-phenylalanine biosynthetic process (0.0460) |
|  |  |  |  | Cytochrome complex assembly (0.0460) |

* Copy-number variants are considered gains or losses with respect to the domesticated genome.

** The unaligned regions were only predicted by SyRI.

**Table S3.** **Information of 192 individuals sequenced using tGBS libraries, including population name, geographical coordinates and SRA accession. (within population names: W = wild, D = domesticated)**

| **Individual ID** | **Population name** | **Population number** | **Taxon** | **Latitude** | **Longitude** | **SRA accession** |
| --- | --- | --- | --- | --- | --- | --- |
| M_SON1 | Alamos, Sonora (Outgroup) | 0 | *Cucurbita moschata* (Outgroup) | 27.02694 | -108.93659 | SRR12937368 |
| M_SON2 | Alamos, Sonora (Outgroup) | 0 | *Cucurbita moschata* (Outgroup) | 27.02694 | -108.93659 | SRR12937367 |
| M_SON3 | Alamos, Sonora (Outgroup) | 0 | *Cucurbita moschata* (Outgroup) | 27.02694 | -108.93659 | SRR12937288 |
| M_SON4 | Alamos, Sonora (Outgroup) | 0 | *Cucurbita moschata* (Outgroup) | 27.02694 | -108.93659 | SRR12937277 |
| M_SON5 | Alamos, Sonora (Outgroup) | 0 | *Cucurbita moschata* (Outgroup) | 27.02694 | -108.93659 | SRR12937266 |
| M_SON6 | Alamos, Sonora (Outgroup) | 0 | *Cucurbita moschata* (Outgroup) | 27.02694 | -108.93659 | SRR12937255 |
| S_CHIS1 | Jiquipilas, Chiapas (W) | 1 | *Cucurbita argyrosperma* subsp. *sororia* | 16.597486 | -93.626878 | SRR12937244 |
| S_CHIS2 | Jiquipilas, Chiapas (W) | 1 | *Cucurbita argyrosperma* subsp. *sororia* | 16.597486 | -93.626878 | SRR12937233 |
| S_CHIS3 | Jiquipilas, Chiapas (W) | 1 | *Cucurbita argyrosperma* subsp. *sororia* | 16.597486 | -93.626878 | SRR12937222 |
| S_CHIS4 | Jiquipilas, Chiapas (W) | 1 | *Cucurbita argyrosperma* subsp. *sororia* | 16.597486 | -93.626878 | SRR12937211 |
| S_CHIS5 | Jiquipilas, Chiapas (W) | 1 | *Cucurbita argyrosperma* subsp. *sororia* | 16.597486 | -93.626878 | SRR12937366 |
| S_CHIS6 | Jiquipilas, Chiapas (W) | 1 | *Cucurbita argyrosperma* subsp. *sororia* | 16.597486 | -93.626878 | SRR12937355 |
| S_CHIS7 | Jiquipilas, Chiapas (W) | 1 | *Cucurbita argyrosperma* subsp. *sororia* | 16.597486 | -93.626878 | SRR12937200 |
| S_CHIS8 | Jiquipilas, Chiapas (W) | 1 | *Cucurbita argyrosperma* subsp. *sororia* | 16.597486 | -93.626878 | SRR12937189 |
| S_GRO1 | Ometepec, Guerrero (W) | 2 | *Cucurbita argyrosperma* subsp. *sororia* | 16.688139 | -98.406319 | SRR12937178 |
| S_GRO2 | Ometepec, Guerrero (W) | 2 | *Cucurbita argyrosperma* subsp. *sororia* | 16.688139 | -98.406319 | SRR12937335 |
| S_GRO3 | Ometepec, Guerrero (W) | 2 | *Cucurbita argyrosperma* subsp. *sororia* | 16.688139 | -98.406319 | SRR12937324 |
| S_GRO4 | Ometepec, Guerrero (W) | 2 | *Cucurbita argyrosperma* subsp. *sororia* | 16.688139 | -98.406319 | SRR12937313 |
| S_GRO5 | Ometepec, Guerrero (W) | 2 | *Cucurbita argyrosperma* subsp. *sororia* | 16.688139 | -98.406319 | SRR12937302 |
| S_OAX1 | Puerto Escondido, Oaxaca (W) | 3 | *Cucurbita argyrosperma* subsp. *sororia* | 15.918225 | -97.076308 | SRR12937291 |
| S_OAX2 | Puerto Escondido, Oaxaca (W) | 3 | *Cucurbita argyrosperma* subsp. *sororia* | 15.918225 | -97.076308 | SRR12937287 |
| S_OAX3 | Puerto Escondido, Oaxaca (W) | 3 | *Cucurbita argyrosperma* subsp. *sororia* | 15.918225 | -97.076308 | SRR12937286 |
| S_OAX4 | Puerto Escondido, Oaxaca (W) | 3 | *Cucurbita argyrosperma* subsp. *sororia* | 15.918225 | -97.076308 | SRR12937285 |
| S_OAX5 | Puerto Escondido, Oaxaca (W) | 3 | *Cucurbita argyrosperma* subsp. *sororia* | 15.918225 | -97.076308 | SRR12937284 |
| S_OAX6 | Puerto Escondido, Oaxaca (W) | 3 | *Cucurbita argyrosperma* subsp. *sororia* | 15.918225 | -97.076308 | SRR12937283 |
| S_OAX7 | Puerto Escondido, Oaxaca (W) | 3 | *Cucurbita argyrosperma* subsp. *sororia* | 15.918225 | -97.076308 | SRR12937282 |
| S_OAX8 | Puerto Escondido, Oaxaca (W) | 3 | *Cucurbita argyrosperma* subsp. *sororia* | 15.918225 | -97.076308 | SRR12937281 |
| S_OAX9 | Puerto Escondido, Oaxaca (W) | 3 | *Cucurbita argyrosperma* subsp. *sororia* | 15.918225 | -97.076308 | SRR12937280 |
| S_OAX10 | Puerto Escondido, Oaxaca (W) | 3 | *Cucurbita argyrosperma* subsp. *sororia* | 15.918225 | -97.076308 | SRR12937279 |
| S_OAX11 | Puerto Escondido, Oaxaca (W) | 3 | *Cucurbita argyrosperma* subsp. *sororia* | 15.9528333 | -97.0772778 | SRR12937278 |
| S_OAX12 | Puerto Escondido, Oaxaca (W) | 3 | *Cucurbita argyrosperma* subsp. *sororia* | 15.9528333 | -97.0772778 | SRR12937276 |
| S_OAX13 | Puerto Escondido, Oaxaca (W) | 3 | *Cucurbita argyrosperma* subsp. *sororia* | 15.9528333 | -97.0772778 | SRR12937275 |
| S_OAX14 | Puerto Escondido, Oaxaca (W) | 3 | *Cucurbita argyrosperma* subsp. *sororia* | 15.9528333 | -97.0772778 | SRR12937274 |
| S_JAL1 | Jalisco (W) | 4 | *Cucurbita argyrosperma* subsp. *sororia* | 19.682 | -104.333278 | SRR12937273 |
| S_JAL2 | Jalisco (W) | 4 | *Cucurbita argyrosperma* subsp. *sororia* | 19.682 | -104.333278 | SRR12937272 |
| S_JAL3 | Jalisco (W) | 4 | *Cucurbita argyrosperma* subsp. *sororia* | 19.7019583 | -104.204314 | SRR12937271 |
| S_JAL4 | Jalisco (W) | 4 | *Cucurbita argyrosperma* subsp. *sororia* | 19.9005833 | -104.160222 | SRR12937270 |
| S_JAL5 | Jalisco (W) | 4 | *Cucurbita argyrosperma* subsp. *sororia* | 19.9005833 | -104.160222 | SRR12937269 |
| S_JAL6 | Jalisco (W) | 4 | *Cucurbita argyrosperma* subsp. *sororia* | 19.9005833 | -104.160222 | SRR12937268 |
| S_JAL7 | Jalisco (W) | 4 | *Cucurbita argyrosperma* subsp. *sororia* | 19.9005833 | -104.160222 | SRR12937267 |
| S_JAL8 | Jalisco (W) | 4 | *Cucurbita argyrosperma* subsp. *sororia* | 19.6997222 | -104.203056 | SRR12937265 |
| S_JAL9 | Jalisco (W) | 4 | *Cucurbita argyrosperma* subsp. *sororia* | 19.6997222 | -104.203056 | SRR12937264 |
| S_JAL10 | Jalisco (W) | 4 | *Cucurbita argyrosperma* subsp. *sororia* | 19.6997222 | -104.203056 | SRR12937263 |
| S_JAL11 | Jalisco (W) | 4 | *Cucurbita argyrosperma* subsp. *sororia* | 19.8753889 | -104.072333 | SRR12937262 |
| S_JAL12 | Jalisco (W) | 4 | *Cucurbita argyrosperma* subsp. *sororia* | 19.9123056 | -104.116833 | SRR12937261 |
| S_JAL13 | Jalisco (W) | 4 | *Cucurbita argyrosperma* subsp. *sororia* | 19.9123056 | -104.116833 | SRR12937260 |
| S_JAL14 | Jalisco (W) | 4 | *Cucurbita argyrosperma* subsp. *sororia* | 19.9123056 | -104.116833 | SRR12937259 |
| S_JAL15 | Jalisco (W) | 4 | *Cucurbita argyrosperma* subsp. *sororia* | 19.9123056 | -104.116833 | SRR12937258 |
| S_JAL16 | Jalisco (W) | 4 | *Cucurbita argyrosperma* subsp. *sororia* | 19.9600833 | -104.037472 | SRR12937257 |
| S_JAL17 | Jalisco (W) | 4 | *Cucurbita argyrosperma* subsp. *sororia* | 19.9574722 | -103.988389 | SRR12937256 |
| S_JAL18 | Jalisco (W) | 4 | *Cucurbita argyrosperma* subsp. *sororia* | 19.8753889 | -104.072333 | SRR12937254 |
| S_JAL19 | Jalisco (W) | 4 | *Cucurbita argyrosperma* subsp. *sororia* | 19.8356111 | -104.081417 | SRR12937253 |
| S_JAL20 | Jalisco (W) | 4 | *Cucurbita argyrosperma* subsp. *sororia* | 19.8356111 | -104.081417 | SRR12937252 |
| S_JAL21 | Jalisco (W) | 4 | *Cucurbita argyrosperma* subsp. *sororia* | 19.6909722 | -104.360833 | SRR12937251 |
| S_JAL22 | Jalisco (W) | 4 | *Cucurbita argyrosperma* subsp. *sororia* | 19.6909722 | -104.360833 | SRR12937250 |
| S_JAL23 | Jalisco (W) | 4 | *Cucurbita argyrosperma* subsp. *sororia* | 19.6909722 | -104.360833 | SRR12937249 |
| A_TLAP1 | Tlapehuala, Guerrero (D) | 5 | *Cucurbita argyrosperma* subsp. *argyrosperma* | 18.2416667 | -100.534722 | SRR12937248 |
| A_TLAP2 | Tlapehuala, Guerrero (D) | 5 | *Cucurbita argyrosperma* subsp. *argyrosperma* | 18.2416667 | -100.534722 | SRR12937247 |
| A_TLAP3 | Tlapehuala, Guerrero (D) | 5 | *Cucurbita argyrosperma* subsp. *argyrosperma* | 18.2416667 | -100.534722 | SRR12937246 |
| A_TLAP4 | Tlapehuala, Guerrero (D) | 5 | *Cucurbita argyrosperma* subsp. *argyrosperma* | 18.2416667 | -100.534722 | SRR12937245 |
| A_TLAP5 | Tlapehuala, Guerrero (D) | 5 | *Cucurbita argyrosperma* subsp. *argyrosperma* | 18.2416667 | -100.534722 | SRR12937243 |
| A_TLAP6 | Tlapehuala, Guerrero (D) | 5 | *Cucurbita argyrosperma* subsp. *argyrosperma* | 18.2416667 | -100.534722 | SRR12937242 |
| A_TLAP7 | Tlapehuala, Guerrero (D) | 5 | *Cucurbita argyrosperma* subsp. *argyrosperma* | 18.2416667 | -100.534722 | SRR12937241 |
| A_TLAP8 | Tlapehuala, Guerrero (D) | 5 | *Cucurbita argyrosperma* subsp. *argyrosperma* | 18.2416667 | -100.534722 | SRR12937240 |
| A_TLAP9 | Tlapehuala, Guerrero (D) | 5 | *Cucurbita argyrosperma* subsp. *argyrosperma* | 18.2416667 | -100.534722 | SRR12937239 |
| A_TLAP10 | Tlapehuala, Guerrero (D) | 5 | *Cucurbita argyrosperma* subsp. *argyrosperma* | 18.2416667 | -100.534722 | SRR12937238 |
| A_JAL1 | Jalisco (D) | 6 | *Cucurbita argyrosperma* subsp. *argyrosperma* | 19.7019583 | -104.204314 | SRR12937237 |
| A_JAL2 | Jalisco (D) | 6 | *Cucurbita argyrosperma* subsp. *argyrosperma* | 19.7019583 | -104.204314 | SRR12937236 |
| A_JAL3 | Jalisco (D) | 6 | *Cucurbita argyrosperma* subsp. *argyrosperma* | 19.6997222 | -104.203056 | SRR12937235 |
| A_JAL4 | Jalisco (D) | 6 | *Cucurbita argyrosperma* subsp. *argyrosperma* | 19.6997222 | -104.203056 | SRR12937234 |
| A_JAL5 | Jalisco (D) | 6 | *Cucurbita argyrosperma* subsp. *argyrosperma* | 19.8753889 | -104.072333 | SRR12937232 |
| A_JAL6 | Jalisco (D) | 6 | *Cucurbita argyrosperma* subsp. *argyrosperma* | 19.8753889 | -104.072333 | SRR12937231 |
| A_JAL7 | Jalisco (D) | 6 | *Cucurbita argyrosperma* subsp. *argyrosperma* | 19.9600833 | -104.037472 | SRR12937230 |
| A_JAL8 | Jalisco (D) | 6 | *Cucurbita argyrosperma* subsp. *argyrosperma* | 19.8714722 | -104.217333 | SRR12937229 |
| A_JAL9 | Jalisco (D) | 6 | *Cucurbita argyrosperma* subsp. *argyrosperma* | 19.8714722 | -104.217333 | SRR12937228 |
| A_JAL10 | Jalisco (D) | 6 | *Cucurbita argyrosperma* subsp. *argyrosperma* | 19.9574722 | -103.988389 | SRR12937227 |
| A_JAL11 | Jalisco (D) | 6 | *Cucurbita argyrosperma* subsp. *argyrosperma* | 19.8356111 | -104.081417 | SRR12937226 |
| A_JAL12 | Jalisco (D) | 6 | *Cucurbita argyrosperma* subsp. *argyrosperma* | 19.8356111 | -104.081417 | SRR12937225 |
| A_JAL13 | Jalisco (D) | 6 | *Cucurbita argyrosperma* subsp. *argyrosperma* | 19.8356111 | -104.081417 | SRR12937224 |
| A_JAL14 | Jalisco (D) | 6 | *Cucurbita argyrosperma* subsp. *argyrosperma* | 19.8356111 | -104.081417 | SRR12937223 |
| A_JAL15 | Jalisco (D) | 6 | *Cucurbita argyrosperma* subsp. *argyrosperma* | 19.8356111 | -104.081417 | SRR12937221 |
| A_JAL16 | Jalisco (D) | 6 | *Cucurbita argyrosperma* subsp. *argyrosperma* | 19.8356111 | -104.081417 | SRR12937220 |
| A_JAL17 | Jalisco (D) | 6 | *Cucurbita argyrosperma* subsp. *argyrosperma* | 19.8356111 | -104.081417 | SRR12937219 |
| A_BAD1 | Badiraguato, Sinaloa (D) | 7 | *Cucurbita argyrosperma* subsp. *argyrosperma* | 25.359173 | -107.558408 | SRR12937218 |
| A_MTP1 | Matlalapa, Guerrero (D) | 8 | *Cucurbita argyrosperma* subsp. *argyrosperma* | 17.5971639 | -99.4579917 | SRR12937217 |
| A_MTP2 | Matlalapa, Guerrero (D) | 8 | *Cucurbita argyrosperma* subsp. *argyrosperma* | 17.5971639 | -99.4579917 | SRR12937216 |
| A_MTP3 | Matlalapa, Guerrero (D) | 8 | *Cucurbita argyrosperma* subsp. *argyrosperma* | 17.5971639 | -99.4579917 | SRR12937215 |
| A_MTP4 | Matlalapa, Guerrero (D) | 8 | *Cucurbita argyrosperma* subsp. *argyrosperma* | 17.5971639 | -99.4579917 | SRR12937214 |
| A_MTP5 | Matlalapa, Guerrero (D) | 8 | *Cucurbita argyrosperma* subsp. *argyrosperma* | 17.5971639 | -99.4579917 | SRR12937213 |
| A_MTP6 | Matlalapa, Guerrero (D) | 8 | *Cucurbita argyrosperma* subsp. *argyrosperma* | 17.5971639 | -99.4579917 | SRR12937212 |
| A_MTP7 | Matlalapa, Guerrero (D) | 8 | *Cucurbita argyrosperma* subsp. *argyrosperma* | 17.5971639 | -99.4579917 | SRR12937210 |
| A_SAH1 | Sahuayo, Michoacán (D) | 9 | *Cucurbita argyrosperma* subsp. *argyrosperma* | 20.0587194 | -102.716233 | SRR12937209 |
| A_SAH2 | Sahuayo, Michoacán (D) | 9 | *Cucurbita argyrosperma* subsp. *argyrosperma* | 20.0587194 | -102.716233 | SRR12937208 |
| A_SAH3 | Sahuayo, Michoacán (D) | 9 | *Cucurbita argyrosperma* subsp. *argyrosperma* | 20.0587194 | -102.716233 | SRR12937207 |
| A_SAH4 | Sahuayo, Michoacán (D) | 9 | *Cucurbita argyrosperma* subsp. *argyrosperma* | 20.0587194 | -102.716233 | SRR12937206 |
| A_SAH5 | Sahuayo, Michoacán (D) | 9 | *Cucurbita argyrosperma* subsp. *argyrosperma* | 20.0587194 | -102.716233 | SRR12937205 |
| A_SAH6 | Sahuayo, Michoacán (D) | 9 | *Cucurbita argyrosperma* subsp. *argyrosperma* | 20.0587194 | -102.716233 | SRR12937204 |
| A_SAH7 | Sahuayo, Michoacán (D) | 9 | *Cucurbita argyrosperma* subsp. *argyrosperma* | 20.0587194 | -102.716233 | SRR12937203 |
| A_SAH8 | Sahuayo, Michoacán (D) | 9 | *Cucurbita argyrosperma* subsp. *argyrosperma* | 20.0587194 | -102.716233 | SRR12937202 |
| A_SAH9 | Sahuayo, Michoacán (D) | 9 | *Cucurbita argyrosperma* subsp. *argyrosperma* | 20.0587194 | -102.716233 | SRR12937201 |
| A_SAH10 | Sahuayo, Michoacán (D) | 9 | *Cucurbita argyrosperma* subsp. *argyrosperma* | 20.0587194 | -102.716233 | SRR12937365 |
| A_SAL1 | Salamanca, Guanajuato (D) | 10 | *Cucurbita argyrosperma* subsp. *argyrosperma* | 20.5205778 | -101.190992 | SRR12937364 |
| A_SJI1 | San José Iturbide, Guanajuato (D) | 10 | *Cucurbita argyrosperma* subsp. *argyrosperma* | 20.9988889 | -100.385 | SRR12937363 |
| A_NAY1 | Tepic, Nayarit (D) | 11 | *Cucurbita argyrosperma* subsp. *argyrosperma* | 21.519956 | -104.893423 | SRR12937362 |
| A_NAY2 | Tepic, Nayarit (D) | 11 | *Cucurbita argyrosperma* subsp. *argyrosperma* | 21.519956 | -104.893423 | SRR12937361 |
| A_NAY3 | Tepic, Nayarit (D) | 11 | *Cucurbita argyrosperma* subsp. *argyrosperma* | 21.519956 | -104.893423 | SRR12937360 |
| A_NAY4 | Tepic, Nayarit (D) | 11 | *Cucurbita argyrosperma* subsp. *argyrosperma* | 21.519956 | -104.893423 | SRR12937359 |
| F_PLAT1 | El Platanar, Sinaloa (feral) | 12 | feral individual | 24.0303667 | -106.432561 | SRR12937358 |
| F_PLAT2 | El Platanar, Sinaloa (feral) | 12 | feral individual | 24.0303667 | -106.432561 | SRR12937357 |
| F_PLAT3 | El Platanar, Sinaloa (feral) | 12 | feral individual | 24.0303667 | -106.432561 | SRR12937356 |
| F_PLAT4 | El Platanar, Sinaloa (feral) | 12 | feral individual | 24.0303667 | -106.432561 | SRR12937354 |
| F_PLAT5 | El Platanar, Sinaloa (feral) | 12 | feral individual | 24.0303667 | -106.432561 | SRR12937353 |
| F_PLAT6 | El Platanar, Sinaloa (feral) | 12 | feral individual | 24.0303667 | -106.432561 | SRR12937352 |
| F_PLAT7 | El Platanar, Sinaloa (feral) | 12 | feral individual | 24.0303667 | -106.432561 | SRR12937351 |
| F_CUL1 | Culiacán, Sinaloa (feral) | 13 | feral individual | 24.817335 | -107.416667 | SRR12937350 |
| F_CUL2 | Culiacán, Sinaloa (feral) | 13 | feral individual | 24.817335 | -107.416667 | SRR12937349 |
| F_CUL3 | Culiacán, Sinaloa (feral) | 13 | feral individual | 24.817335 | -107.416667 | SRR12937348 |
| F_CUL4 | Culiacán, Sinaloa (feral) | 13 | feral individual | 24.817335 | -107.416667 | SRR12937347 |
| F_CUL5 | Culiacán, Sinaloa (feral) | 13 | feral individual | 24.817335 | -107.416667 | SRR12937346 |
| A_CHOI1 | Choix, Sinaloa (D) | 14 | *Cucurbita argyrosperma* subsp. *argyrosperma* | 26.5967833 | -108.335581 | SRR12937345 |
| A_CHOI2 | Choix, Sinaloa (D) | 14 | *Cucurbita argyrosperma* subsp. *argyrosperma* | 26.5967833 | -108.335581 | SRR12937199 |
| A_CHOI3 | Choix, Sinaloa (D) | 14 | *Cucurbita argyrosperma* subsp. *argyrosperma* | 26.5967833 | -108.335581 | SRR12937198 |
| A_CHOI4 | Choix, Sinaloa (D) | 14 | *Cucurbita argyrosperma* subsp. *argyrosperma* | 26.5967833 | -108.335581 | SRR12937197 |
| A_CHOI5 | Choix, Sinaloa (D) | 14 | *Cucurbita argyrosperma* subsp. *argyrosperma* | 26.5967833 | -108.335581 | SRR12937196 |
| A_YEC1 | Yecora, Sonora (D) | 15 | *Cucurbita argyrosperma* subsp. *argyrosperma* | 28.3720417 | -108.926986 | SRR12937195 |
| A_YEC2 | Yecora, Sonora (D) | 15 | *Cucurbita argyrosperma* subsp. *argyrosperma* | 28.3720417 | -108.926986 | SRR12937194 |
| A_YEC3 | Yecora, Sonora (D) | 15 | *Cucurbita argyrosperma* subsp. *argyrosperma* | 28.3720417 | -108.926986 | SRR12937193 |
| A_YEC4 | Yecora, Sonora (D) | 15 | *Cucurbita argyrosperma* subsp. *argyrosperma* | 28.3720417 | -108.926986 | SRR12937192 |
| A_YEC5 | Yecora, Sonora (D) | 15 | *Cucurbita argyrosperma* subsp. *argyrosperma* | 28.3720417 | -108.926986 | SRR12937191 |
| A_YEC6 | Yecora, Sonora (D) | 15 | *Cucurbita argyrosperma* subsp. *argyrosperma* | 28.3720417 | -108.926986 | SRR12937190 |
| F_ONAV1 | Onavas, Sonora (feral) | 16 | feral individual | 28.533333 | -109.583333 | SRR12937188 |
| F_ONAV2 | Onavas, Sonora (feral) | 16 | feral individual | 28.533333 | -109.583333 | SRR12937187 |
| F_ONAV3 | Onavas, Sonora (feral) | 16 | feral individual | 28.533333 | -109.583333 | SRR12937186 |
| F_ONAV4 | Onavas, Sonora (feral) | 16 | feral individual | 28.533333 | -109.583333 | SRR12937185 |
| F_ONAV5 | Onavas, Sonora (feral) | 16 | feral individual | 28.533333 | -109.583333 | SRR12937184 |
| F_ONAV6 | Onavas, Sonora (feral) | 16 | feral individual | 28.533333 | -109.583333 | SRR12937183 |
| F_ONAV7 | Onavas, Sonora (feral) | 16 | feral individual | 28.533333 | -109.583333 | SRR12937182 |
| A_DGO1 | Durango, Durango (D) | 17 | *Cucurbita argyrosperma* subsp. *argyrosperma* | 24.066667 | -104.583333 | SRR12937181 |
| A_TEH1 | Tehuantepec, Oaxaca (D) | 18 | *Cucurbita argyrosperma* subsp. *argyrosperma* | 16.3328306 | -95.2330361 | SRR12937180 |
| A_TEH2 | Tehuantepec, Oaxaca (D) | 18 | *Cucurbita argyrosperma* subsp. *argyrosperma* | 16.3328306 | -95.2330361 | SRR12937179 |
| A_TEH3 | Tehuantepec, Oaxaca (D) | 18 | *Cucurbita argyrosperma* subsp. *argyrosperma* | 16.3328306 | -95.2330361 | SRR12937177 |
| A_TEH4 | Tehuantepec, Oaxaca (D) | 18 | *Cucurbita argyrosperma* subsp. *argyrosperma* | 16.3328306 | -95.2330361 | SRR12937344 |
| A_TEH5 | Tehuantepec, Oaxaca (D) | 18 | *Cucurbita argyrosperma* subsp. *argyrosperma* | 16.3328306 | -95.2330361 | SRR12937343 |
| A_TEH6 | Tehuantepec, Oaxaca (D) | 18 | *Cucurbita argyrosperma* subsp. *argyrosperma* | 16.3328306 | -95.2330361 | SRR12937342 |
| A_TEH7 | Tehuantepec, Oaxaca (D) | 18 | *Cucurbita argyrosperma* subsp. *argyrosperma* | 16.3328306 | -95.2330361 | SRR12937341 |
| A_ONAV1 | Onavas, Sonora (D) | 19 | *Cucurbita argyrosperma* subsp. *argyrosperma* | 28.533333 | -109.583333 | SRR12937340 |
| A_ONAV2 | Onavas, Sonora (D) | 19 | *Cucurbita argyrosperma* subsp. *argyrosperma* | 28.533333 | -109.583333 | SRR12937339 |
| A_ONAV3 | Onavas, Sonora (D) | 19 | *Cucurbita argyrosperma* subsp. *argyrosperma* | 28.533333 | -109.583333 | SRR12937338 |
| A_ONAV4 | Onavas, Sonora (D) | 19 | *Cucurbita argyrosperma* subsp. *argyrosperma* | 28.533333 | -109.583333 | SRR12937337 |
| A_ONAV5 | Onavas, Sonora (D) | 19 | *Cucurbita argyrosperma* subsp. *argyrosperma* | 28.533333 | -109.583333 | SRR12937336 |
| A_ONAV6 | Onavas, Sonora (D) | 19 | *Cucurbita argyrosperma* subsp. *argyrosperma* | 28.533333 | -109.583333 | SRR12937334 |
| A_VER1 | Tihuatlán, Veracruz (D) | 20 | *Cucurbita argyrosperma* subsp. *argyrosperma* | 20.7200667 | -97.5395028 | SRR12937333 |
| A_VER2 | Tihuatlán, Veracruz (D) | 20 | *Cucurbita argyrosperma* subsp. *argyrosperma* | 20.7200667 | -97.5395028 | SRR12937332 |
| A_VER3 | Tihuatlán, Veracruz (D) | 20 | *Cucurbita argyrosperma* subsp. *argyrosperma* | 20.7200667 | -97.5395028 | SRR12937331 |
| A_VER4 | Tihuatlán, Veracruz (D) | 20 | *Cucurbita argyrosperma* subsp. *argyrosperma* | 20.7200667 | -97.5395028 | SRR12937330 |
| A_VER5 | Tihuatlán, Veracruz (D) | 20 | *Cucurbita argyrosperma* subsp. *argyrosperma* | 20.7200667 | -97.5395028 | SRR12937329 |
| A_PAL1 | Palenque, Chiapas (D) | 21 | *Cucurbita argyrosperma* subsp. *argyrosperma* | 17.5128139 | -91.9877611 | SRR12937328 |
| A_PAL2 | Palenque, Chiapas (D) | 21 | *Cucurbita argyrosperma* subsp. *argyrosperma* | 17.5128139 | -91.9877611 | SRR12937327 |
| A_PAL3 | Palenque, Chiapas (D) | 21 | *Cucurbita argyrosperma* subsp. *argyrosperma* | 17.5128139 | -91.9877611 | SRR12937326 |
| A_PAL4 | Palenque, Chiapas (D) | 21 | *Cucurbita argyrosperma* subsp. *argyrosperma* | 17.5128139 | -91.9877611 | SRR12937325 |
| A_PAL5 | Palenque, Chiapas (D) | 21 | *Cucurbita argyrosperma* subsp. *argyrosperma* | 17.5128139 | -91.9877611 | SRR12937323 |
| A_PAL6 | Palenque, Chiapas (D) | 21 | *Cucurbita argyrosperma* subsp. *argyrosperma* | 17.5128139 | -91.9877611 | SRR12937322 |
| A_SLP1 | Tanquián, San Luis Potosí (D) | 22 | *Cucurbita argyrosperma* subsp. *argyrosperma* | 22.1154861 | -101.009331 | SRR12937321 |
| A_SLP2 | Tanquián, San Luis Potosí (D) | 22 | *Cucurbita argyrosperma* subsp. *argyrosperma* | 22.1154861 | -101.009331 | SRR12937320 |
| A_SLP3 | Tanquián, San Luis Potosí (D) | 22 | *Cucurbita argyrosperma* subsp. *argyrosperma* | 22.1154861 | -101.009331 | SRR12937319 |
| A_SLP4 | Tanquián, San Luis Potosí (D) | 22 | *Cucurbita argyrosperma* subsp. *argyrosperma* | 22.1154861 | -101.009331 | SRR12937318 |
| A_SLP5 | Tanquián, San Luis Potosí (D) | 22 | *Cucurbita argyrosperma* subsp. *argyrosperma* | 22.1154861 | -101.009331 | SRR12937317 |
| A_SLP6 | Tanquián, San Luis Potosí (D) | 22 | *Cucurbita argyrosperma* subsp. *argyrosperma* | 22.1154861 | -101.009331 | SRR12937316 |
| A_CHAMP1 | Champotón, Campeche (D) | 23 | *Cucurbita argyrosperma* subsp. *argyrosperma* | 19.5011556 | -90.4613778 | SRR12937315 |
| A_CHAMP2 | Champotón, Campeche (D) | 23 | *Cucurbita argyrosperma* subsp. *argyrosperma* | 19.5011556 | -90.4613778 | SRR12937314 |
| A_CHAMP3 | Champotón, Campeche (D) | 23 | *Cucurbita argyrosperma* subsp. *argyrosperma* | 19.5011556 | -90.4613778 | SRR12937312 |
| A_CHAMP4 | Champotón, Campeche (D) | 23 | *Cucurbita argyrosperma* subsp. *argyrosperma* | 19.5011556 | -90.4613778 | SRR12937311 |
| A_CHAMP5 | Champotón, Campeche (D) | 23 | *Cucurbita argyrosperma* subsp. *argyrosperma* | 19.5011556 | -90.4613778 | SRR12937310 |
| A_MIXT1 | Mixtepec, Oaxaca (D) | 24 | *Cucurbita argyrosperma* subsp. *argyrosperma* | 15.95875 | -97.0849167 | SRR12937309 |
| A_MIXT2 | Mixtepec, Oaxaca (D) | 24 | *Cucurbita argyrosperma* subsp. *argyrosperma* | 15.95875 | -97.0849167 | SRR12937308 |
| A_MIXT3 | Mixtepec, Oaxaca (D) | 24 | *Cucurbita argyrosperma* subsp. *argyrosperma* | 15.95875 | -97.0849167 | SRR12937307 |
| A_MIXT4 | Mixtepec, Oaxaca (D) | 24 | *Cucurbita argyrosperma* subsp. *argyrosperma* | 15.95875 | -97.0849167 | SRR12937306 |
| A_MIXT5 | Mixtepec, Oaxaca (D) | 24 | *Cucurbita argyrosperma* subsp. *argyrosperma* | 15.95875 | -97.0849167 | SRR12937305 |
| A_MIXT6 | Mixtepec, Oaxaca (D) | 24 | *Cucurbita argyrosperma* subsp. *argyrosperma* | 15.95875 | -97.0849167 | SRR12937304 |
| A_EK1 | Ek Balam, Yucatan (D) | 25 | *Cucurbita argyrosperma* subsp. *argyrosperma* | 20.9166667 | -87.9166667 | SRR12937303 |
| A_EK2 | Ek Balam, Yucatan (D) | 25 | *Cucurbita argyrosperma* subsp. *argyrosperma* | 20.9166667 | -87.9166667 | SRR12937301 |
| A_EK3 | Ek Balam, Yucatan (D) | 25 | *Cucurbita argyrosperma* subsp. *argyrosperma* | 20.9166667 | -87.9166667 | SRR12937300 |
| A_EK4 | Ek Balam, Yucatan (D) | 25 | *Cucurbita argyrosperma* subsp. *argyrosperma* | 20.9166667 | -87.9166667 | SRR12937299 |
| A_EK5 | Ek Balam, Yucatan (D) | 25 | *Cucurbita argyrosperma* subsp. *argyrosperma* | 20.9166667 | -87.9166667 | SRR12937298 |
| A_EK6 | Ek Balam, Yucatan (D) | 25 | *Cucurbita argyrosperma* subsp. *argyrosperma* | 20.9166667 | -87.9166667 | SRR12937297 |
| A_CHAN1 | Chan Santa Cruz, Quintana Roo (D) | 26 | *Cucurbita argyrosperma* subsp. *argyrosperma* | 19.3670639 | -88.3328917 | SRR12937296 |
| A_CHAN2 | Chan Santa Cruz, Quintana Roo (D) | 26 | *Cucurbita argyrosperma* subsp. *argyrosperma* | 19.3670639 | -88.3328917 | SRR12937295 |
| A_CHAN3 | Chan Santa Cruz, Quintana Roo (D) | 26 | *Cucurbita argyrosperma* subsp. *argyrosperma* | 19.3670639 | -88.3328917 | SRR12937294 |
| A_CHAN4 | Chan Santa Cruz, Quintana Roo (D) | 26 | *Cucurbita argyrosperma* subsp. *argyrosperma* | 19.3670639 | -88.3328917 | SRR12937293 |
| A_CHAN5 | Chan Santa Cruz, Quintana Roo (D) | 26 | *Cucurbita argyrosperma* subsp. *argyrosperma* | 19.3670639 | -88.3328917 | SRR12937292 |
| A_CHAN6 | Chan Santa Cruz, Quintana Roo (D) | 26 | *Cucurbita argyrosperma* subsp. *argyrosperma* | 19.3670639 | -88.3328917 | SRR12937290 |
| A_CHAN7 | Chan Santa Cruz, Quintana Roo (D) | 26 | *Cucurbita argyrosperma* subsp. *argyrosperma* | 19.3670639 | -88.3328917 | SRR12937289 |

**Table S4. Average genetic diversity of the wild, domesticated and feral populations of *Cucurbita argyrosperma* using 2,861 unlinked SNPs predicted with the domesticated (D) reference genome, and 1,771 unlinked SNPs predicted with the wild (W) reference genome.**

| **Taxon** | ***N*_ind_** | ***N*_pop_** | ***H*_O_ (Var)** | | ***H*_E_ (Var)** | | ***π* (Var)** | | ***F*_IS_ (Var)** | |
| --- | --- | --- | --- | --- | --- | --- | --- | --- | --- | --- |
|  |  |  | **D** | **W** | **D** | **W** | **D** | **W** | **D** | **W** |
| *Cucurbita argyrosperma* subsp. *sororia* | 44 | 4 | 0.098 (0.017) | 0.089 (0.016) | 0.096 (0.014) | 0.094 (0.015) | 0.098 (0.015) | 0.096 (0.015) | 0.011 (0.027) | 0.044 (0.037) |
| *Cucurbita argyrosperma* subsp. *argyrosperma* | 109 | 19 | 0.094 (0.012) | 0.095 (0.015) | 0.094 (0.010) | 0.097 (0.012) | 0.095 (0.010) | 0.098 (0.012) | 0.034 (0.030) | 0.067 (0.038) |
| feral populations | 14 | 3 | 0.102 (0.032) | 0.102 (0.035) | 0.088 (0.019) | 0.089 (0.022) | 0.094 (0.023) | 0.096 (0.026) | -0.015 (0.022) | -0.011 (0.026) |
| *Cucurbita moschata* (outgroup) | 5 | 1 | 0.077 (0.042) | 0.080 (0.047) | 0.058 (0.019) | 0.058 (0.021) | 0.068 (0.029) | 0.069 (0.032) | -0.017 (0.017) | -0.020 (0.019) |

(*N*_ind_ = number of individuals, *N*_pop_ = number of populations, *H*_O_ = observed heterozygosity, *H*_E_ = expected heterozygosity, *π* = nucleotide diversity, *F*_IS_ = inbreeding coefficient, Var = variance)

**Table S5. Genetic diversity of each wild, domesticated and feral population of *Cucurbita argyrosperma* using 2,861 unlinked SNPs (r2 < 0.25, MAF > 1%).**

| **Population name** | **Population number** | ***N*** | ***H*_O_ (Var)** | ***H*_E_ (Var)** | ***π* (Var)** | ***F*_IS_ (Var)** |
| --- | --- | --- | --- | --- | --- | --- |
| *Cucurbita moschata* (Outgroup) | 0 | 5 | 0.077 (0.042) | 0.058 (0.019) | 0.068 (0.029) | -0.018 (0.017) |
| Jiquipilas, Chiapas (W) | 1 | 8 | 0.091 (0.039) | 0.073 (0.020) | 0.082 (0.027) | -0.020 (0.017) |
| Ometepec, Guerrero (W) | 2 | 5 | 0.089 (0.038) | 0.076 (0.021) | 0.088 (0.030) | 0.001 (0.027) |
| Puerto Escondido, Oaxaca (W) | 3 | 14 | 0.094 (0.031) | 0.079 (0.018) | 0.085 (0.022) | -0.021 (0.017) |
| Jalisco (W) | 4 | 17 | 0.115 (0.033) | 0.099 (0.020) | 0.106 (0.024) | -0.020 (0.020) |
| Tlapehuala, Guerrero (D) | 5 | 10 | 0.098 (0.029) | 0.086 (0.018) | 0.093 (0.022) | -0.010 (0.023) |
| Jalisco (D) | 6 | 13 | 0.099 (0.025) | 0.090 (0.017) | 0.096 (0.020) | -0.006 (0.022) |
| Badiraguato, Sinaloa (D) | 7 | 1 | 0.095 (0.086) | 0.047 (0.021) | 0.095 (0.086) | 0.000 (0.000) |
| Matlalapa, Guerrero (D) | 8 | 7 | 0.092 (0.029) | 0.081 (0.018) | 0.090 (0.023) | -0.006 (0.017) |
| Sahuayo, Michoacán (D) | 9 | 10 | 0.101 (0.027) | 0.091 (0.018) | 0.097 (0.021) | -0.008 (0.022) |
| Salamanca, Guanajuato (D) | 10 | 1 | 0.087 (0.079) | 0.043 (0.020) | 0.087 (0.079) | 0.000 (0.000) |
| Tepic, Nayarit (D) | 11 | 4 | 0.089 (0.044) | 0.067 (0.020) | 0.083 (0.034) | -0.012 (0.012) |
| El Platanar, Sinaloa (feral) | 12 | 4 | 0.107 (0.062) | 0.074 (0.024) | 0.097 (0.046) | -0.019 (0.018) |
| Culiacán, Sinaloa (feral) | 13 | 3 | 0.099 (0.064) | 0.066 (0.023) | 0.094 (0.053) | -0.010 (0.014) |
| Choix, Sinaloa (D) | 14 | 3 | 0.101 (0.060) | 0.069 (0.023) | 0.096 (0.050) | -0.008 (0.014) |
| Yecora, Sonora (D) | 15 | 6 | 0.104 (0.040) | 0.081 (0.020) | 0.092 (0.027) | -0.025 (0.013) |
| Onavas, Sonora (feral) | 16 | 7 | 0.101 (0.044) | 0.077 (0.022) | 0.088 (0.029) | -0.026 (0.015) |
| Durango, Durango (D) | 17 | 1 | 0.076 (0.071) | 0.038 (0.017) | 0.076 (0.071) | 0.000 (0.000) |
| Tehuantepec, Oaxaca (D) | 18 | 7 | 0.085 (0.032) | 0.071 (0.018) | 0.079 (0.023) | -0.014 (0.014) |
| Onavas, Sonora (D) | 19 | 6 | 0.089 (0.040) | 0.067 (0.018) | 0.078 (0.026) | -0.025 (0.013) |
| Tihuatlán, Veracruz (D) | 20 | 5 | 0.088 (0.041) | 0.069 (0.020) | 0.082 (0.030) | -0.011 (0.018) |
| Palenque, Chiapas (D) | 21 | 5 | 0.089 (0.035) | 0.072 (0.019) | 0.083 (0.026) | -0.013 (0.015) |
| Tanquián, San Luis Potosí (D) | 22 | 6 | 0.090 (0.033) | 0.074 (0.019) | 0.084 (0.025) | -0.013 (0.013) |
| Champotón, Campeche (D) | 23 | 5 | 0.096 (0.039) | 0.076 (0.020) | 0.090 (0.030) | -0.011 (0.016) |
| Mixtepec, Oaxaca (D) | 24 | 6 | 0.101 (0.038) | 0.081 (0.020) | 0.092 (0.027) | -0.020 (0.016) |
| Ek Balam, Yucatan (D) | 25 | 6 | 0.098 (0.041) | 0.075 (0.020) | 0.086 (0.027) | -0.026 (0.014) |
| Chan Santa Cruz, Quintana Roo (D) | 26 | 7 | 0.089 (0.032) | 0.073 (0.018) | 0.081 (0.022) | -0.018 (0.016) |

(*N* = sample size, *H*_O_ = observed heterozygosity, *H*_E_ = expected heterozygosity, *π* = nucleotide diversity, *F*_IS_ = inbreeding coefficient, Var = variance) (within population names: W = wild, D = domesticated)

**Table S6. Results of ABBA-BABA test to detect introgression using 11,498,421 variants between *C. argyrosperma* subsp. *sororia* (P1), *C. argyrosperma* subp. *argyrosperma* (P2) and *C. moschata* (P3), while using *C. okeechobeensis* subsp. *martinezii* as an outgroup.**

| **P1** | **P2** | **P3** | **AABB sites** | **ABBA sites** | **BABA sites** | ***D*-statistic** | ***p*-value** | ***f*_G_** |
| --- | --- | --- | --- | --- | --- | --- | --- | --- |
| *C. argyrosperma* subsp. *sororia* | *C. argyrosperma* subsp. *argyrosperma* | *C. moschata* | 652029 | 90253.8 | 81267 | 0.0523945 | 0.001414 | 0.0106867 |

**Table S7.** **Candidate genes containing at least one outlier SNP (predicted by at least two tests) within their inner structure (introns, exons, UTRs). The direction of selection was inferred according to the ancestral and derived allelic state for each outlier SNP. (AED = Annotation Edit Distance; GO ID = Gene Ontology ID)**

| **Direction of selection** | **Gene ID (domesticated genome)** | **Gene ID (wild genome)** | **Chromosome location** | **Functional annotation against SwissProt** | **AED** | **GO ID** |
| --- | --- | --- | --- | --- | --- | --- |
| domesticated populations | Carg02896 | Csor.00g176320 | Chr09 | Similar to *AKT1* Potassium channel *AKT1* (*Arabidopsis thaliana*) | 0.14 | GO:0005216, GO:0006811, GO:0016020, GO:0055085 |
| domesticated populations | Carg04908 | Csor.00g009170 | Chr08 | Similar to *RPS15AE* 40S ribosomal protein S15a-5 (*Arabidopsis thaliana*) | 0.11 | GO:0003735, GO:0005840, GO:0006412 |
| domesticated populations | Carg07327 | Csor.00g196260 | Chr01 | Similar to *dusA* tRNA-dihydrouridine(20/20a) synthase (*Vibrio vulnificus*) | 0.27 | GO:0008033, GO:0017150, GO:0050660, GO:0055114 |
| domesticated populations | Carg09511 | Csor.00g083590 | Chr17 | Similar to *IAA27* Auxin-responsive protein *IAA27* (*Arabidopsis thaliana*) | 0.14 | NA |
| domesticated populations | Carg12845 | Csor.00g037030 | Chr09 | Similar to *GDPDL4* Glycerophosphodiester phosphodiesterase *GDPDL4* (*Arabidopsis thaliana*) | 0.1 | GO:0006629, GO:0008081 |
| domesticated populations | Carg13010 | Csor.00g214890 | Chr10 | Similar to At4g29530 Thiamine phosphate phosphatase-like protein (*Arabidopsis thaliana*) | 0.11 | GO:0016791 |
| domesticated populations | Carg13432 | Csor.00g005000 | Chr08 | Similar to *efr3b* Protein *EFR3* homolog B (*Danio rerio*) | 0.16 | NA |
| domesticated populations | Carg14512 | Csor.00g157750 | Chr18 | Similar to *MKP1* Protein-tyrosine-phosphatase *MKP1* (*Arabidopsis thaliana*) | 0.01 | GO:0008138, GO:0016311 |
| domesticated populations | Carg20078 | Csor.00g227200 | Chr09 | Similar to *ABCE2* ABC transporter E family member 2 (*Arabidopsis thaliana*) | 0.12 | GO:0005524, GO:0016887 |
| domesticated populations | Carg20623 | Csor.00g016670 | Chr13 | Protein of unknown function | 0.03 | GO:0005515 |
| domesticated populations | Carg21521 | Csor.00g160720 | Chr13 | Similar to *SNI1* Negative regulator of systemic acquired resistance *SNI1* (*Arabidopsis thaliana*) | 0.19 | NA |
| domesticated populations | Carg23167 | Csor.00g003720 | Chr08 | Similar to *CES101* G-type lectin S-receptor-like serine/threonine-protein kinase *CES101* (*Arabidopsis thaliana*) | 0.04 | GO:0004672, GO:0004674, GO:0005524, GO:0006468 |
| domesticated populations | Carg26378 | Csor.00g260430 | Chr11 | Similar to *DLO1* Protein *DMR6*-LIKE OXYGENASE 1 (*Arabidopsis thaliana*) | 0.16 | GO:0016491, GO:0055114 |
| wild populations | Carg00678 | Csor.00g059490 | Chr03 | Similar to *CSI1* Protein CELLULOSE SYNTHASE INTERACTIVE 1 (*Arabidopsis thaliana*) | 0.06 | GO:0005515 |
| wild populations | Carg00942 | Csor.00g292650 | Chr06 | Similar to *CTN* Cactin (*Arabidopsis thaliana*) | 0.14 | GO:0005515 |
| wild populations | Carg01177 | Csor.00g247900 | Chr06 | Protein of unknown function | 0.13 | GO:0071816 |
| wild populations | Carg01823 | Csor.00g164870 | Chr04 | Similar to *PP2AA2* Serine/threonine-protein phosphatase 2A 65 kDa regulatory subunit A beta isoform (*Arabidopsis thaliana*) | 0.19 | GO:0005515 |
| wild populations | Carg02996 | NA | Chr09 | Similar to At5g49980 Transport inhibitor response 1-like protein (*Arabidopsis thaliana*) | 0.02 | GO:0005515 |
| wild populations | Carg04587 | Csor.00g123000 | Chr04 | Similar to At3g53190 Probable pectate lyase 12 (*Arabidopsis thaliana*) | 0.06 | NA |
| wild populations | Carg04921 | Csor.00g009020 | Chr08 | Similar to *ATX2* Histone-lysine N-methyltransferase *ATX2* (*Arabidopsis thaliana*) | 0.23 | GO:0005515, GO:0005634 |
| wild populations | Carg05727 | Csor.00g172100 | Chr16 | Similar to *KIN7G* Kinesin-like protein *KIN-7G* (*Arabidopsis thaliana*) | 0.11 | GO:0003777, GO:0005524, GO:0007018, GO:0008017 |
| wild populations | Carg06997 | Csor.00g080170 | Chr01 | Similar to *IREH1* Probable serine/threonine protein kinase *IREH1* (*Arabidopsis thaliana*) | 0.08 | GO:0004672, GO:0005524, GO:0006468 |
| wild populations | Carg07232 | Csor.00g265760 | Chr17 | Similar to *HULK3* Protein *HUA2*-LIKE 3 (*Arabidopsis thaliana*) | 0.22 | NA |
| wild populations | Carg07327 | Csor.00g196260 | Chr01 | Similar to *dusA* tRNA-dihydrouridine(20/20a) synthase (*Vibrio vulnificus*) | 0.27 | GO:0008033, GO:0017150, GO:0050660, GO:0055114 |
| wild populations | Carg07889 | Csor.00g113710 | Chr07 | Similar to *SFH9* Phosphatidylinositol/phosphatidylcholine transfer protein *SFH9* (*Arabidopsis thaliana*) | 0.14 | NA |
| wild populations | Carg09452 | Csor.00g084200 | Chr17 | Similar to *ALA4* Probable phospholipid-transporting ATPase 4 (*Arabidopsis thaliana*) | 0.09 | GO:0000166, GO:0000287, GO:0005524, GO:0015914, GO:0016021, GO:0140326 |
| wild populations | Carg10718 | Csor.00g080500 | Chr01 | Similar to *FBL15* F-box/LRR-repeat protein 15 (*Arabidopsis thaliana*) | 0.16 | GO:0005515 |
| wild populations | Carg11621 | Csor.00g072250 | Chr02 | Similar to Zeaxanthin epoxidase, chloroplastic (*Prunus armeniaca*) | 0.12 | GO:0005515, GO:0009507, GO:0009540, GO:0009688, GO:0016020, GO:0055114, GO:0071949 |
| wild populations | Carg12374 | Csor.00g192590 | Chr01 | Similar to *PBL10* Probable serine/threonine-protein kinase *PBL10* (*Arabidopsis thaliana*) | 0.19 | GO:0004672, GO:0006468 |
| wild populations | Carg14512 | Csor.00g157750 | Chr18 | Similar to *MKP1* Protein-tyrosine-phosphatase *MKP1* (*Arabidopsis thaliana*) | 0.01 | GO:0008138, GO:0016311 |
| wild populations | Carg14932 | Csor.00g116960 | Chr07 | Similar to *VPS54* Vacuolar protein sorting-associated protein 54, chloroplastic (*Arabidopsis thaliana*) | 0.26 | GO:0005515, GO:0008080, GO:0042147 |
| wild populations | Carg15904 | Csor.00g236940 | Chr08 | Similar to *SGS3* Protein SUPPRESSOR OF GENE SILENCING 3 homolog (*Oryza sativa* subsp. *indica*) | 0.13 | GO:0031047 |
| wild populations | Carg15929 | Csor.00g237260 | Chr08 | Similar to At5g19025 Uncharacterized protein At5g19025 (*Arabidopsis thaliana*) | 0.28 | NA |
| wild populations | Carg18944 | Csor.00g084780 | Chr17 | Similar to *ATL3* RING-H2 finger protein *ATL3* (*Arabidopsis thaliana*) | 0.01 | NA |
| wild populations | Carg22232 | Csor.00g242160 | Chr16 | Similar to *POP1* Ribonucleases P/MRP protein subunit *POP1* (*Homo sapiens*) | 0.1 | NA |
| wild populations | Carg23772 | Csor.00g220140 | Chr04 | Similar to *SAC3A* *SAC3* family protein A (*Arabidopsis thaliana*) | 0.14 | NA |
| wild populations | Carg23802 | NA | Chr14 | Similar to At1g06840 Probable LRR receptor-like serine/threonine-protein kinase At1g06840 (*Arabidopsis thaliana*) | 0.19 | GO:0005515 |
| wild populations | Carg24812 | NA | Chr16 | Similar to *PBL23* Probable serine/threonine-protein kinase *PBL23* (*Arabidopsis thaliana*) | 0.07 | GO:0004672, GO:0005524, GO:0006468 |
| wild populations | Carg25546 | Csor.00g002770 | Chr17 | Similar to *TMKL1* Putative kinase-like protein *TMKL1* (*Arabidopsis thaliana*) | 0.3 | GO:0004672, GO:0005515, GO:0006468 |
| wild populations | Carg25639 | Csor.00g029150 | Chr01 | Similar to *OVA7* Serine--tRNA ligase, chloroplastic/mitochondrial (*Arabidopsis thaliana*) | 0.12 | GO:0000166, GO:0004812, GO:0004828, GO:0005524, GO:0006418, GO:0006434 |
| wild populations | Carg26784 | NA | Chr15 | Protein of unknown function | 0.02 | NA |
| wild populations | Carg26826 | Csor.00g030720 | Chr16 | Similar to *PA200* Proteasome activator subunit 4 (*Arabidopsis thaliana*) | 0.14 | NA |
| wild populations | Carg27622 | Csor.00g103900 | Chr20 | Similar to *SUMO2* Small ubiquitin-related modifier 2 (*Arabidopsis thaliana*) | 0.32 | NA |
| wild populations | Carg_TCONS_00026631 | NA | Chr13 | Long noncoding RNA | NA | NA |
| ABBA sites | Carg07674 | Csor.00g064770 | Chr13 | Similar to *apaG* Protein *ApaG* (*Magnetospirillum magneticum*) | 0.21 | GO:0005515 |
| ABBA sites | Carg26784 | NA | Chr15 | Protein of unknown function | 0.02 | NA |
| ABBA sites | Carg26826 | Csor.00g030720 | Chr16 | Similar to *PA200* Proteasome activator subunit 4 (*Arabidopsis thaliana*) | 0.14 | NA |
| BABA sites | Carg04520 | Csor.00g122340 | Chr04 | Similar to *RBCMT* Ribulose-1,5 bisphosphate carboxylase/oxygenase large subunit N-methyltransferase, chloroplastic (*Nicotiana tabacum*) | 0.23 | GO:0005515 |
| BABA sites | Carg20078 | Csor.00g227200 | Chr09 | Similar to *ABCE2* ABC transporter E family member 2 (*Arabidopsis thaliana*) | 0.12 | GO:0005524, GO:0016887 |
| BABA sites | Carg21397 | Csor.00g161930 | Chr13 | Similar to *AGD12* ADP-ribosylation factor GTPase-activating protein AGD12 (*Arabidopsis thaliana*) | 0.16 | GO:0005096 |
| BABA sites | Carg22875 | Csor.00g267800 | Chr15 | Similar to *SPBC3E7*.09 Uncharacterized protein slp1 (Schizosaccharomyces pombe) | 0.11 | NA |


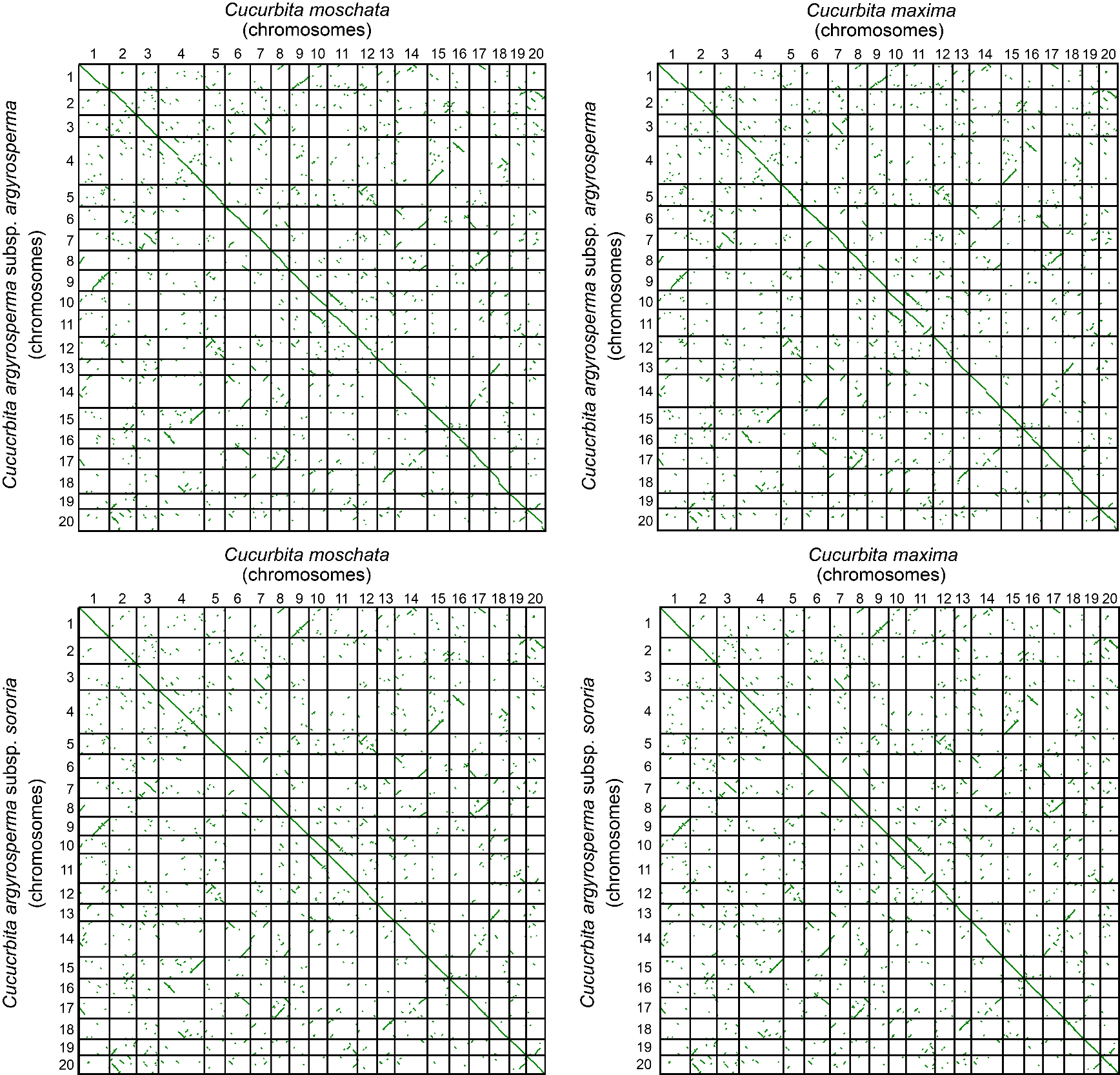
**Supplementary Figures**

**Fig. S1.** **Synteny dot plots between the chromosome-level genome assemblies of *Cucurbita argyrosperma* subsp. *argyrosperma* and *C. argyrosperma* subsp. *sororia* against the reference genomes of *C. moschata* and *C. maxima* (Sun *et al*., 2017).** Most of the chromosomes show chromosome-wide homoeologous pairs within the genome assembly, which have been previously attributed to a whole-genome duplication event in the *Cucurbita* genus.


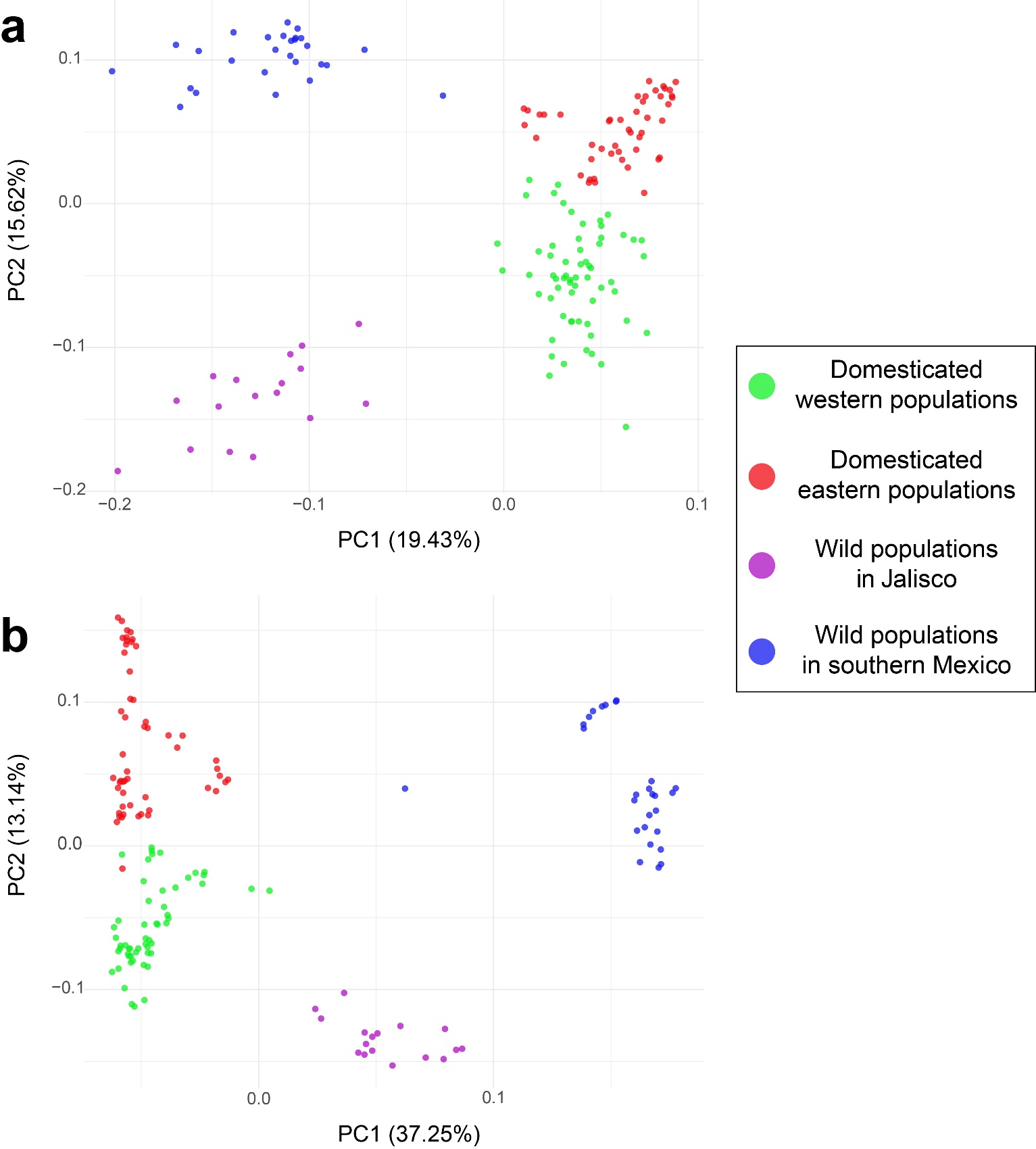


**Fig. S2. Principal Component Analysis (PCA) plots from data of *C. argyrosperma* subsp. *argyrosperma* (domesticated) and *C. argyrosperma* subsp. *sororia* (wild) populations.** The first two components were plotted for **a** the SNPs dataset used for demographic analyses (2,861 SNPs,153 individuals) and for **b** the SNP dataset used for the selection scans (10,617 SNPs, 153 individuals).


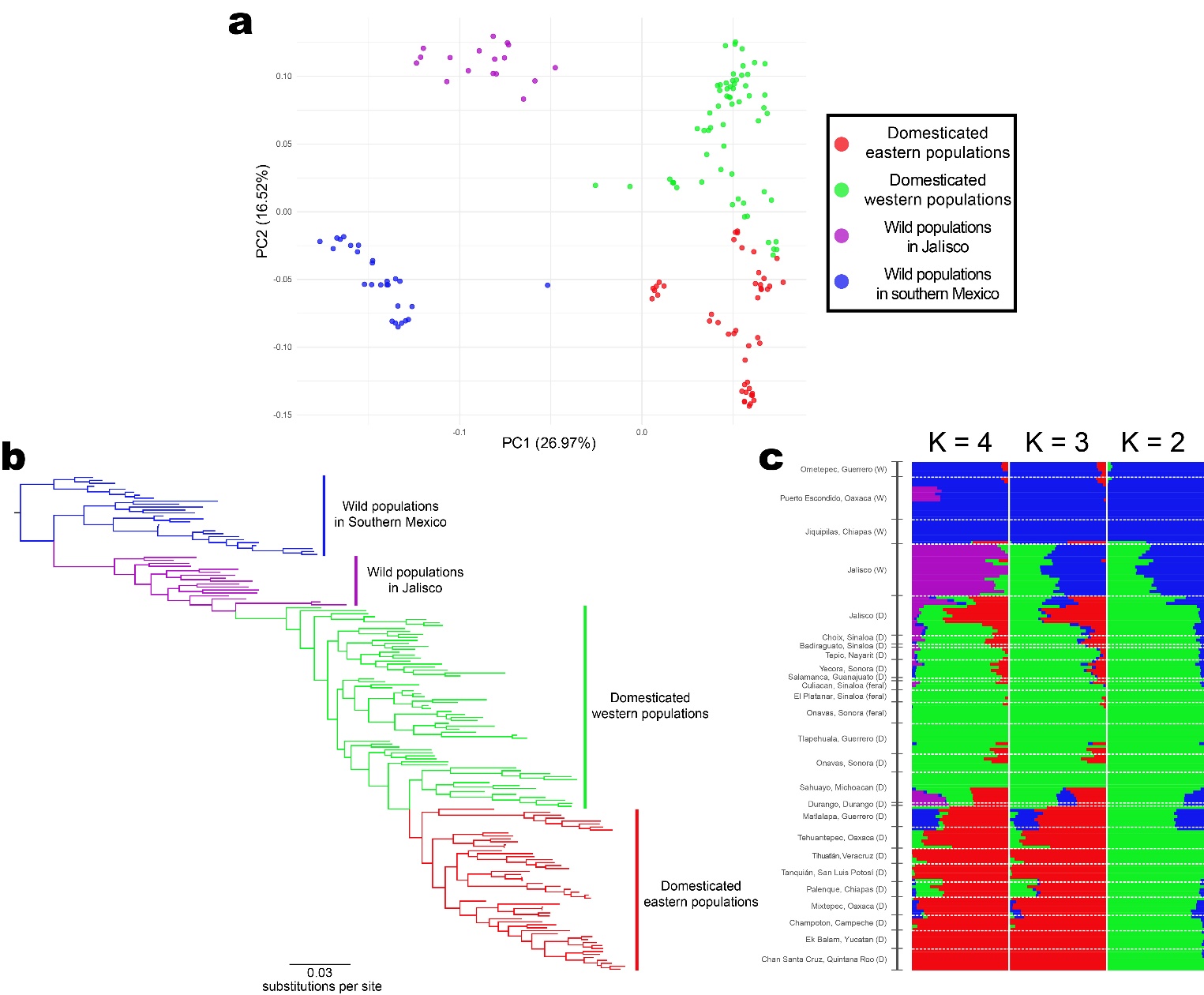


**Fig. S3.** **Genetic structure and phylogenetic relationships between the wild and domesticated populations of *Cucurbita argyrosperma* retrieved from an alternative SNP dataset.** The filtering step of 50% missing data was applied separately for the domesticated and wild populations. We also omitted the filter of the Hardy-Weinberg equilibrium exact test. **a** Principal component analysis. **b** Maximum Likelihood tree. **c** ADMIXTURE analysis using K values ranging from 2 to 4.


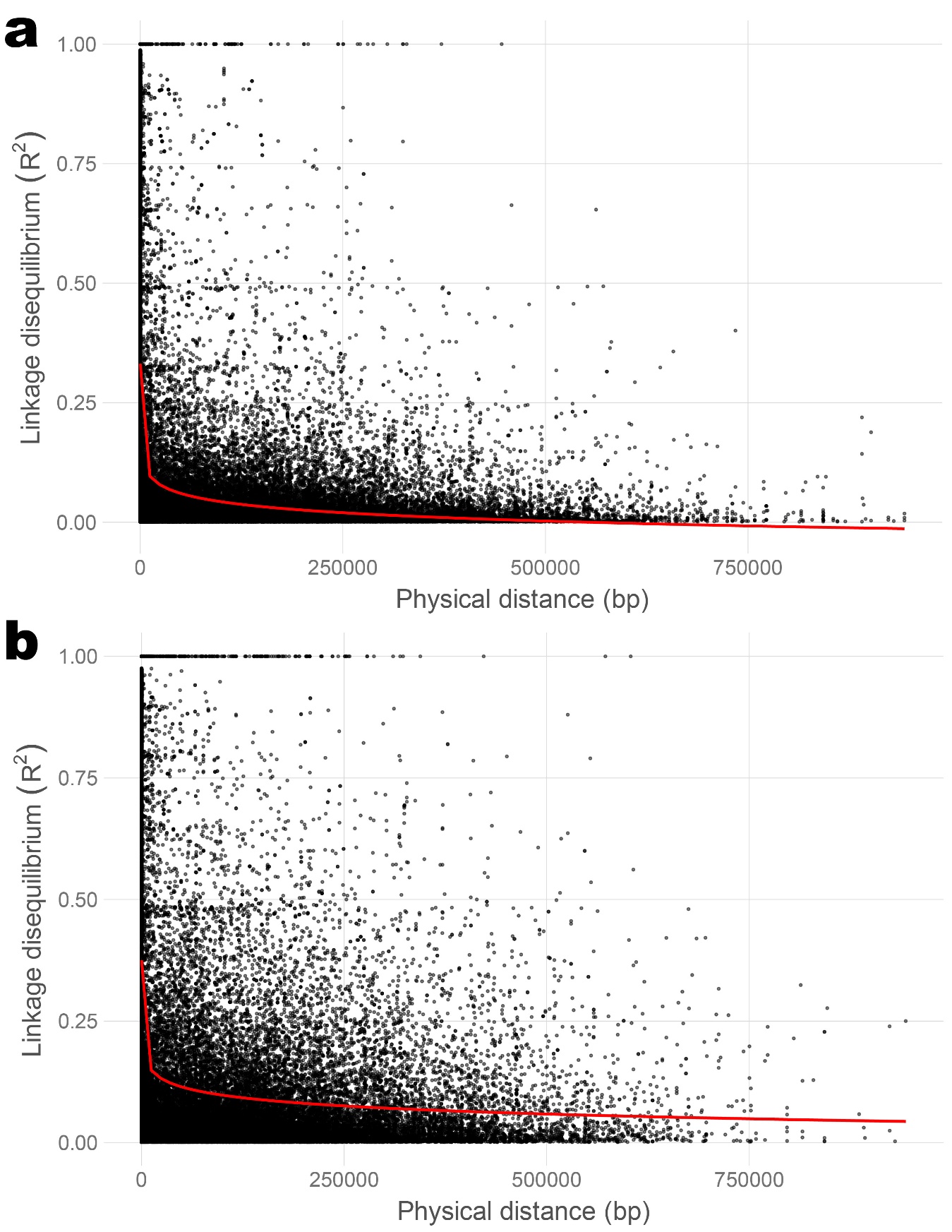


**Fig. S4. Linkage disequilibrium (LD) decay between the 10,617 SNPs used to perform the selective scans in *C. argyrosperma*.** **a** LD decay in *C. argyrosperma* subsp. *argyrosperma*. **b** LD decay in *C. argyrosperma* subsp. *sororia*.


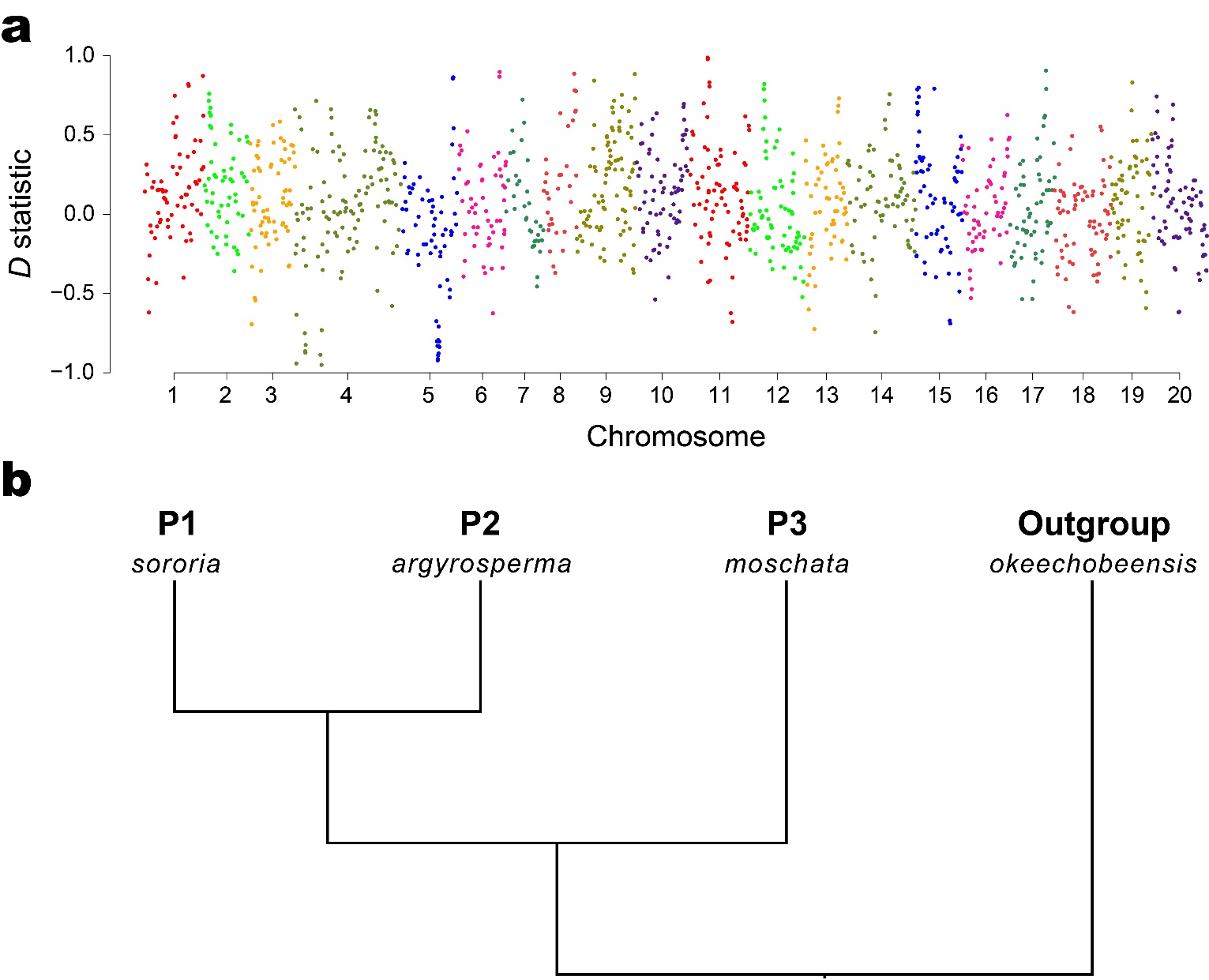


**Fig. S5.** **ABBA-BABA test to detect introgression using 11,498,421 variants between *C. argyrosperma* subsp. *sororia* (P1), *C. argyrosperma* subp. *argyrosperma* (P2) and *C. moschata* (P3), while using *C. okeechobeensis* subsp. *martinezii* as an outgroup.** **a** Manhattan plot of the *D*-statistic throughout the genome using 500 SNP windows with a step size of 250 SNPs. **b** Phylogenetic topology used to perform the ABBA-BABA test, as reconstructed by Dsuite.

**Supplementary Data**

**Data S1: Coalescent simulation parameters of domestication in Jalisco with constant gene flow (Model 1).**

*//Number of population samples (demes)*

*3 samples to simulate :*

*//Population effective sizes (number of genes)*

*WILD_JALISCO_NPOP*

*WILD_SOUTH_NPOP*

*DOMESTICATED_NPOP*

*//Sample sizes*

*12*

*26*

*26*

*//Growth rates : negative growth implies population expansion*

*0*

*0*

*0*

*//Number of migration matrices: 0 implies no migration between demes*

*3*

*//Migration 0*

*0.0000 MIG10 MIG20*

*MIG01 0.0000 MIG21*

*MIG02 MIG12 0.0000*

*//Migration1*

*0.000 MIG10 0.000*

*MIG01 0.000 0.000*

*0.000 0.000 0.000*

*//Migration2*

*0.000 0.000 0.000*

*0.000 0.000 0.000*

*0.000 0.000 0.000*

*//historical event: time, source, sink, migrants, new size, growth rate, migr. matrix*

*2 historical event*

*TDOMESTICATED 2 0 1 RESDOM 0 1*

*TWILDSOUTH 1 0 1 RESWILDS 0 2*

*//Number of independent loci*

*1 0*

*//Per chromosome: Number of linkage blocks*

*1*

*//per Block: data type, num loci, rec. rate and mut rate + optional parameters*

*FREQ 1 0 2.5e-8 OUTEXP*

**Data S2: Coalescent simulation parameters of domestication in Jalisco with secondary contact (Model 2).**

*//Number of population samples (demes)*

*3 samples to simulate :*

*//Population effective sizes (number of genes)*

*WILD_JALISCO_NPOP*

*WILD_SOUTH_NPOP*

*DOMESTICATED_NPOP*

*//Sample sizes*

*12*

*26*

*26*

*//Growth rates : negative growth implies population expansion*

*0*

*0*

*0*

*//Number of migration matrices: 0 implies no migration between demes*

*3*

*//Migration 0*

*0.0000 MIG10 MIG20*

*MIG01 0.0000 MIG21*

*MIG02 MIG12 0.0000*

*//Migration1*

*0.000 0.000 0.000*

*0.000 0.000 0.000*

*0.000 0.000 0.000*

*//Migration2*

*0.000 0.000 0.000*

*0.000 0.000 0.000*

*0.000 0.000 0.000*

*//historical event: time, source, sink, migrants, new size, growth rate, migr. matrix*

*2 historical event*

*TDOMESTICATED 2 0 1 RESDOM 0 1*

*TWILDSOUTH 1 0 1 RESWILDS 0 2*

*//Number of independent loci*

*1 0*

*//Per chromosome: Number of linkage blocks*

*1*

*//per Block: data type, num loci, rec. rate and mut rate + optional parameters*

*FREQ 1 0 2.5e-8 OUTEXP*

**Data S3: Coalescent simulation parameters of domestication in Jalisco with no gene flow (Model 3).**

*//Number of population samples (demes)*

*3 samples to simulate :*

*//Population effective sizes (number of genes)*

*WILD_JALISCO_NPOP*

*WILD_SOUTH_NPOP*

*DOMESTICATED_NPOP*

*//Sample sizes*

*12*

*26*

*26*

*//Growth rates : negative growth implies population expansion*

*0*

*0*

*0*

*//Number of migration matrices: 0 implies no migration between demes*

*3*

*//Migration 0*

*0.0000 0.0000 0.0000*

*0.0000 0.0000 0.0000*

*0.0000 0.0000 0.0000*

*//Migration1*

*0.0000 0.0000 0.0000*

*0.0000 0.0000 0.0000*

*0.0000 0.0000 0.0000*

*//Migration2*

*0.000 0.000 0.000*

*0.000 0.000 0.000*

*0.000 0.000 0.000*

*//historical event: time, source, sink, migrants, new size, growth rate, migr. matrix*

*2 historical event*

*TDOMESTICATED 2 0 1 RESDOM 0 1*

*TWILDSOUTH 1 0 1 RESWILDS 0 2*

*//Number of independent loci*

*1 0*

*//Per chromosome: Number of linkage blocks*

*1*

*//per Block: data type, num loci, rec. rate and mut rate + optional parameters*

*FREQ 1 0 2.5e-8 OUTEXP*

**Data S4: Coalescent simulation parameters of domestication in southern Mexico with constant gene flow (Model 4).**

*//Number of population samples (demes)*

*3 samples to simulate :*

*//Population effective sizes (number of genes)*

*WILD_SOUTH_NPOP*

*WILD_JALISCO_NPOP*

*DOMESTICATED_NPOP*

*//Sample sizes*

*12*

*26*

*26*

*//Growth rates : negative growth implies population expansion*

*0*

*0*

*0*

*//Number of migration matrices: 0 implies no migration between demes*

*3*

*//Migration 0*

*0.0000 MIG10 MIG20*

*MIG01 0.0000 MIG21*

*MIG02 MIG12 0.0000*

*//Migration1*

*0.000 MIG10 0.000*

*MIG01 0.000 0.000*

*0.000 0.000 0.000*

*//Migration2*

*0.000 0.000 0.000*

*0.000 0.000 0.000*

*0.000 0.000 0.000*

*//historical event: time, source, sink, migrants, new size, growth rate, migr. matrix*

*2 historical event*

*TDOMESTICATED 2 0 1 RESDOM 0 1*

*TWILDJALISCO 1 0 1 RESWILDJ 0 2*

*//Number of independent loci*

*1 0*

*//Per chromosome: Number of linkage blocks*

*1*

*//per Block: data type, num loci, rec. rate and mut rate + optional parameters*

*FREQ 1 0 2.5e-8 OUTEXP*

**Data S5: Coalescent simulation parameters of domestication in southern Mexico with secondary contact (Model 5).**

*//Number of population samples (demes)*

*3 samples to simulate :*

*//Population effective sizes (number of genes)*

*WILD_SOUTH_NPOP*

*WILD_JALISCO_NPOP*

*DOMESTICATED_NPOP*

*//Sample sizes*

*12*

*26*

*26*

*//Growth rates : negative growth implies population expansion*

*0*

*0*

*0*

*//Number of migration matrices: 0 implies no migration between demes*

*3*

*//Migration 0*

*0.0000 MIG10 MIG20*

*MIG01 0.0000 MIG21*

*MIG02 MIG12 0.0000*

*//Migration1*

*0.000 0.000 0.000*

*0.000 0.000 0.000*

*0.000 0.000 0.000*

*//Migration2*

*0.000 0.000 0.000*

*0.000 0.000 0.000*

*0.000 0.000 0.000*

*//historical event: time, source, sink, migrants, new size, growth rate, migr. matrix*

*2 historical event*

*TDOMESTICATED 2 0 1 RESDOM 0 1*

*TWILDJALISCO 1 0 1 RESWILDJ 0 2*

*//Number of independent loci*

*1 0*

*//Per chromosome: Number of linkage blocks*

*1*

*//per Block: data type, num loci, rec. rate and mut rate + optional parameters*

*FREQ 1 0 2.5e-8 OUTEXP*

**Data S6: Coalescent simulation parameters of domestication in southern Mexico with no gene flow (Model 6).**

*//Number of population samples (demes)*

*3 samples to simulate :*

*//Population effective sizes (number of genes)*

*WILD_SOUTH_NPOP*

*WILD_JALISCO_NPOP*

*DOMESTICATED_NPOP*

*//Sample sizes*

*12*

*26*

*26*

*//Growth rates : negative growth implies population expansion*

*0*

*0*

*0*

*//Number of migration matrices: 0 implies no migration between demes*

*3*

*//Migration 0*

*0.0000 0.0000 0.0000*

*0.0000 0.0000 0.0000*

*0.0000 0.0000 0.0000*

*//Migration1*

*0.0000 0.0000 0.0000*

*0.0000 0.0000 0.0000*

*0.0000 0.0000 0.0000*

*//Migration2*

*0.000 0.000 0.000*

*0.000 0.000 0.000*

*0.000 0.000 0.000*

*//historical event: time, source, sink, migrants, new size, growth rate, migr. matrix*

*2 historical event*

*TDOMESTICATED 2 0 1 RESDOM 0 1*

*TWILDJALISCO 1 0 1 RESWILDJ 0 2*

*//Number of independent loci*

*1 0*

*//Per chromosome: Number of linkage blocks*

*1*

*//per Block: data type, num loci, rec. rate and mut rate + optional parameters*

*FREQ 1 0 2.5e-8 OUTEXP*
